# Cumulative muscle mass and blood pressure but not fat mass drives arterial stiffness and carotid intima-media thickness progression in the young population and is unrelated to vascular organ damage

**DOI:** 10.1038/s41440-022-01065-1

**Published:** 2022-10-14

**Authors:** Andrew O. Agbaje, Alan R. Barker, Tomi-Pekka Tuomainen

**Affiliations:** 1 Institute of Public Health and Clinical Nutrition, School of Medicine, Faculty of Health Sciences, University of Eastern Finland, Kuopio, Finland; 2 Children’s Health and Exercise Research Centre, Sport and Health Sciences, University of Exeter, Exeter, UK

**Keywords:** Obesity, Vascular aging, Childhood, Longitudinal study, Hypertension

## Abstract

We examined the longitudinal associations of fat mass, lean mass, and blood pressure (BP) from childhood through young adulthood with changes in carotid-femoral pulse wave velocity (cfPWV), a measure of arterial stiffness, and carotid intima-media thickness (cIMT). We included 3863 participants from the Avon Longitudinal Study of Parents and Children birth cohort. Fat mass and lean mass, measured by dual-energy X-ray absorptiometry, and BP were measured at ages 9, 17 and 24 years and classified into low, moderate, and high tertiles. cfPWV and cIMT were measured at 17 and 24 years of age. Associations were examined via linear mixed effect models and adjusted for cardiometabolic and lifestyle factors. Among 1720 [44.5%] male and 2143 [55.5%] female participants, cumulative high exposures to lean mass (effect estimate 0.006 m/s [95% CI 0.001 to 0.010; *p* = 0.022]), systolic BP (0.013 m/s [0.009 to 0.017; *p* < 0.0001]) and diastolic BP (0.023 m/s [0.019 to 0.027; *p* < 0.0001]) from 9–24 years of age were positively associated with the 7-year increase in cfPWV. Persistent high exposures to lean mass (0.012 mm; [0.008 to 0.016; *p* < 0.0001]), body mass index (0.007 mm [0.003 to 0.011; *p* = 0.001]), and systolic BP (0.010 mm; [0.006 to 0.014; *p* < 0.0001]) from ages 9–24 years were positively associated with thicker cIMT at 17–24 years of age. Total fat and trunk fat mass from childhood had no association with cfPWV or cIMT progression. In conclusion, increased lean mass and BP but not fat mass from childhood drives arterial remodeling in young adulthood.

## Graphical abstract

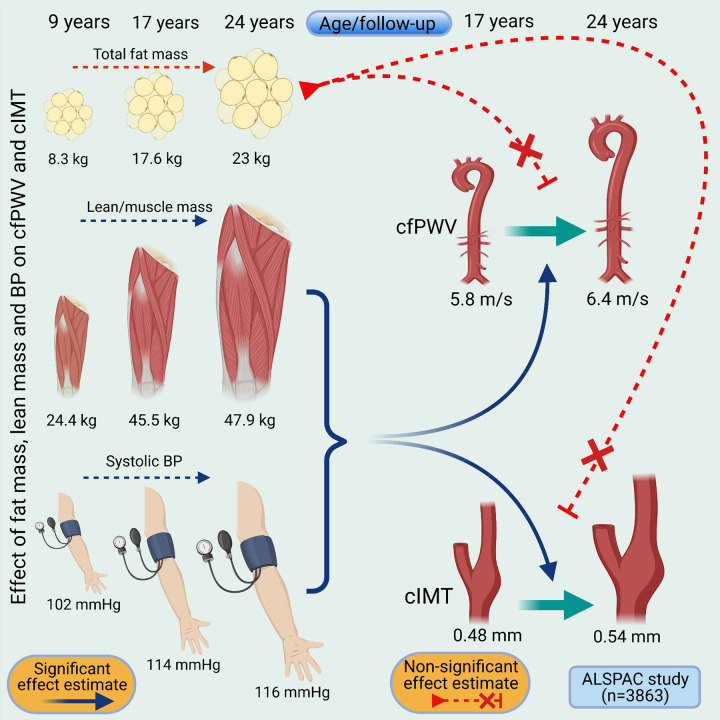

## Introduction

Carotid intima-media thickness (cIMT) and carotid-femoral pulse wave velocity (cfPWV), a measure of arterial stiffness, are important markers of subclinical atherosclerotic cardiovascular morbidity and all-cause mortality [1–5]. Considering the global burden of cardiovascular diseases [6], lifetime risk factor management has been recommended for preventing future cardiovascular events [1–3, 7–10]. Cumulative exposures to modifiable lifetime risks such as childhood obesity, inexpensively estimated using body mass index (BMI), have been associated with faster cfPWV and thicker cIMT in children, adolescents, and adults [2, 11, 12]. However, emerging evidence among children and adolescents [3, 4, 7, 8] suggests that these previous findings [2, 11, 12] might have been inadequately interpreted since BMI poorly discriminates the contribution of fat mass from that of lean mass [13]. Moreover, a Mendelian randomization study found no causal role in the association of BMI with cfPWV and cIMT at age 17 years [8], but more studies are needed that utilized body composition quantified by dual X-ray energy absorptiometry (DEXA), which depicts cardiovascular risk more accurately than traditional anthropometrics [14]. Our present study may clarify whether previous prospective associations of body composition assessed by BMI with vascular structural and functional measures [11, 12] are indeed precursors of arteriosclerotic and atherosclerotic cardiovascular disease risk, early vascular aging, or vascular adaptative responses to increased total and trunk fat mass or lean mass during growth and maturation.

A recent study showed that persistent exposure to high blood pressure (BP), another lifetime modifiable risk, from ages 18–39 years was strongly associated with a 21–37% increased risk of cardiovascular events in late adulthood [15]. However, the study [15] lacked data to investigate this question from childhood, during which period there is limited evidence [10]. Since clinical events or hard outcomes are rare between 9 and 24 years of age, we investigated whether cumulative high exposures to systolic BP and diastolic BP were differently and independently associated with cfPWV and cIMT progression from adolescence through young adulthood. By using data from the Avon Longitudinal Study of Parents and Children (ALSPAC) UK birth cohort, we investigated the prospective associations of cumulative exposures to total and trunk fat mass, lean mass, BMI, and BP from childhood through young adulthood (ages 9–24 years) with cfPWV and cIMT progression from ages 17–24 years.

## Methods

### Study cohort

**Fig. 1:**
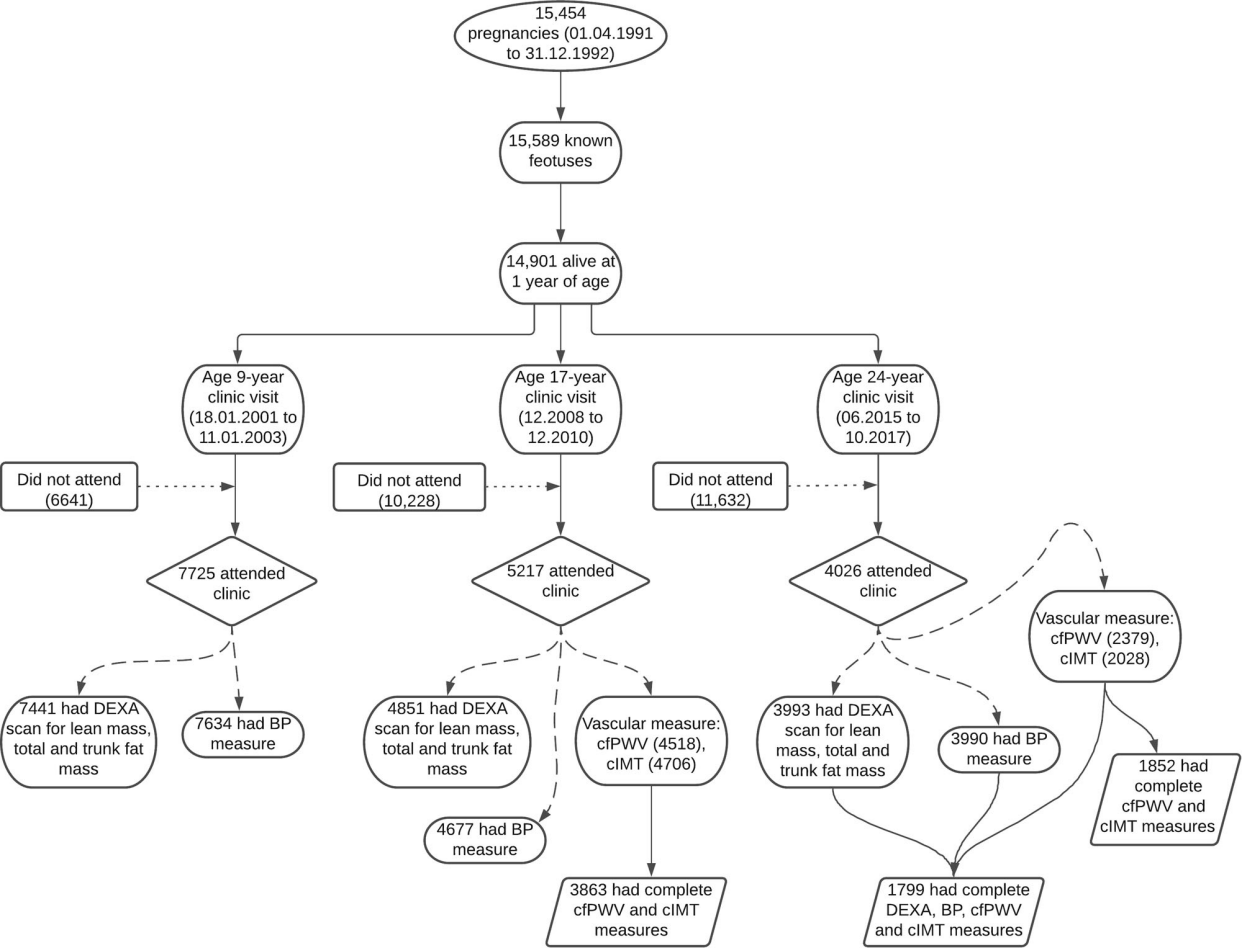
Flowchart of study participants. DEXA, dual-energy X-ray absorptiometry; BP, blood pressure; cfPWV, carotid-femoral pulse wave velocity; cIMT, carotid intima-media thickness. Participants who had complete data for predictors and/or outcomes of interest were included in the analyses

The unabridged method can be found in the supplementary material. Data were from the ALSPAC birth cohort, which investigates factors that influence childhood development and growth. Altogether, 15,454 pregnant women from Avon, southwestern England, UK, who had a total of 15,589 fetuses, were enrolled between April 1, 1991, and December 31, 1992. When the oldest children were approximately 7 years of age, an attempt was made to bolster the initial sample with eligible cases who had failed to join the study originally, resulting in 14,901 children alive at 1 year of age. Regular clinic visits of the children commenced at 7 years of age and are still ongoing. Study data at 24 years were collected and managed using REDCap electronic data capture tools [16]. In the cross-sectional analyses, we included 1799 participants who had complete measurement data for total fat mass, trunk fat mass, lean mass, height, weight, BP, cfPWV, and cIMT during the 24-year follow-up clinic visit. In the prospective analyses, 3863 participants who had complete clinic measurements for cfPWV and cIMT at the 17-year follow-up clinic visit were eligible for analyses (Fig. 1). The demographic characteristics of the excluded individuals were similar to those of individuals included in this study (Supplementary Table 1). Ethical approval for the study was obtained from the ALSPAC Ethics and Law Committee and the Local Research Ethics Committees. Informed consent for the use of data collected via questionnaires and clinics [17–19] was obtained from participants following the recommendations of the ALSPAC Ethics and Law Committee at the time, and the study has therefore been performed in accordance with the ethical standards set forth in the 1964 Declaration of Helsinki and its later amendments. Consent for the use of biological samples was collected in accordance with the Human Tissue Act (2004). Please note that the study website contains details of all the data that are available through a fully searchable data dictionary and variable search tool (http://www.bristol.ac.uk/alspac/researchers/our-data/).

### Anthropometry and body composition

Anthropometry (height and weight) at ages 9 and 17 years was assessed using standard protocols [7]. At 24 years of age, standing height to the nearest millimeter was measured using a Harpenden wall-mounted stadiometer (Holtain Ltd, Crosswell, Crymych, UK). Weight to the nearest 0.1 kg at age 24 years was measured using Tanita TBF-401 (Model A, Tanita Corp., Tokyo, Japan) electronic body composition scales. At ages 9 and 17 years, body composition was assessed using a DEXA scanner (GE Medical Systems, Madison, Wisconsin) as previously described [3, 7]. At 24 years, body composition was measured using a DEXA scanner (Lunar Prodigy software version 15, GE Medical Systems, Madison, Wisconsin) [20]. Repeated DEXA measurements for 122 children were performed on the same day, and the repeatability coefficient (twice the standard deviation of the difference between measurement occasions) for body fat mass was 0.5 kg [3, 7]. We calculated body mass index (BMI) by dividing weight by squared height, total and trunk fat mass indices (FMI) as the ratio of total and trunk fat mass to squared height, and lean mass index (LMI) as the ratio of lean mass to squared height [4]. Participants’ BMIs at ages 17 and 24 years were also categorized as normal weight if <24.99 kg/m^2^ and overweight and obese if >24.99 kg/m^2^.

### Vascular phenotype

At ages 17 and 24 years, cfPWV was computed from pressure waveforms obtained using the Vicorder device (Skidmore Medical, Bristol, UK), while cIMT at 17 and 24 years was assessed by ultrasound using a linear 12-MHz transducer (Vivid7, GE Medical, Chicago, Illinois) and CardioHealth Panasonic and a 13.5 MHz linear array broadband transducer, as previously reported [3, 7, 20]. For our analysis, we computed the mean of the average measurement of the right and left common carotid arteries as cIMT. The age- and sex-specific 90th percentile of cfPWV and cIMT indicates a high risk of subclinical arteriosclerosis and atherosclerosis, respectively.

### Cardiometabolic and lifestyle factors

Pulse rate and BP were measured at ages 9 and 17 years as previously detailed [3, 7]. BP readings at the 24-year clinic visit were taken using an Omron M6 upper arm BP/pulse monitor. Participants at ages 17 and 24 years were categorized as normotensive if systolic BP was <120 mm Hg and as having elevated BP or hypertension when systolic BP was >120 mmHg [9]. Fewer than ten participants received antihypertensive medication at the age of 17 years, but these data were unavailable at 24 years. Using standard protocols, blood samples at ages 9, 17, and 24 years were collected, spun, and frozen at −80 °C, and a detailed assessment of fasting glucose, insulin, high sensitivity C-reactive protein (hsCRP), low-density lipoprotein cholesterol (LDL-C), high-density lipoprotein cholesterol, and triglycerides has been reported (coefficient of variation was <5%) [3, 20, 21].

Questionnaires to assess smoking behavior were administered at the 17-year and 24-year clinic visits [7, 20, 21]. The participants were asked whether they smoked in the last 30 days, smoked a whole cigarette, and smoked every day, along with their frequency of use. At the 17-year clinic visit, participants were briefly asked about their personal and family (mother, father, and siblings) medical history, such as a history of hypertension, diabetes, high cholesterol, and vascular disease [20, 21]. Physical activity at the age of 24 years was assessed with an ActiGraph GT3X + accelerometer device worn for four consecutive days using an established cutoff [22].

Extensive details on multiple imputations [23, 24] are provided in the supplemental methods and results (Supplementary Tables 2–6) [23]. Imputed results of cross-sectional and longitudinal analyses are presented in the main article, while for sensitivity analysis, the nonimputed results are presented in Supplementary Tables 7–9.

### Statistical analysis

The participants’ descriptive characteristics are reported in detail in the supplementary appendix.

We examined the separate cross-sectional associations of total fat mass, total FMI, trunk fat mass, trunk FMI, lean mass, LMI, BMI, or BP (predictors) with cfPWV and cIMT (outcomes) at age 24 years using multiple linear regression analyses. We investigated across-mean differences using generalized linear multivariable-adjusted analyses with Sidak correction for multiple comparisons, while a one-way analysis of variance was used to examine the linear trend.

To examine the separate cumulative effects of total fat mass, total FMI, trunk fat mass, trunk FMI, lean mass, LMI, BMI, or BP measured from ages 9–24 years on the 7-year change in cfPWV and cIMT from adolescence to adulthood, we used linear mixed-effect models. The estimates quantify the effect of cumulative exposures to the predictors on the outcome variables. We classified the predictors based on age- and population-specific tertiles (3 equal distribution), low, moderate, and high categories, and computed two models per outcome. Model 1 was adjusted for sex; age at baseline; time, i.e., difference in years (continuous variable) between baseline measure at age 9 years and that at age 24 years follow-up; cardiometabolic factors such as systolic BP, LDL-C, and hsCRP at ages 9, 17, and 24 years; fasting blood glucose at ages 17 and 24 years; and fat mass and/or lean mass at ages 9, 17, and 24 years depending on the predictor. Model 2 further adjusted for lifestyle factors, i.e., moderate to vigorous physical activity at age 24 years, smoking status at ages 17 and 24 years, and family history of hypertension/diabetes/high cholesterol/vascular disease. We did not adjust the models for heart rate due to high collinearity (*r* = 0.87) with systolic BP. Additionally, height was not adjusted for because we observed near-perfect collinearity with lean mass (*r* = 0.95). For sensitivity analyses, we also presented results based on BMI categories of normal weight status (<24.99 kg/m^2^) and overweight and obesity status (>24.99 kg/m^2^) at the age of 24 years in the Supplementary appendix.

Furthermore, we examined the separate effect of cumulative exposures to the predictor on cfPWV and cIMT at 24 years of age. We also investigated the role of vascular organ damage as a surrogate for subclinical atherosclerosis (≥90th percentile cIMT) and subclinical arteriosclerosis (≥90th percentile cfPWV) on the cumulative effect of total fat mass, lean mass, and systolic blood pressure on cfPWV and cIMT progression with a similar adjustment strategy, using the linear mixed-effect model as detailed above. All covariates were selected based on previous studies [3, 4, 7, 8, 14, 15, 20, 21, 25]. All statistical analyses were performed using SPSS statistics software, Version 27.0 (IBM Corp, Armonk, NY, USA).

## Results

### Study population and characteristics

Of 14,901 children who were alive at 1 year of age, 7725 children participated in the 9-year follow-up, 5217 adolescents participated in the 17-year follow-up, and 4026 young adults participated in the 24-year follow-up clinic visits (Fig. 1). Of these, 3863 participants at the 17-year follow-up and 1852 participants at the 24-year follow-up had complete cfPWV and cIMT measurements. At the 24-year follow-up clinic visit, 1799 participants had complete measurements for fat mass, lean mass, systolic and diastolic BP, cfPWV, and cIMT. The female participants consistently had higher total fat mass, trunk fat mass, total FMI, and trunk FMI and lower lean mass and LMI at the ages of 9, 17, and 24 years than the males (Table 1). However, the significant difference in BMI between sexes at the ages of 9 and 17 years was attenuated at the age of 24 years. Female participants consistently had lower systolic BP, cfPWV, and cIMT at the 17- and 24-year follow-ups than males. As expected, overweight and obese participants either at age 17 or 24 years had worse cardiovascular and metabolic profiles than normal-weight individuals (Supplementary Tables 10 and 11). The prevalence of elevated systolic BP ( >120 mm Hg) and hypertension at 17 years and 24 years was 26.1% and 32.6%, respectively (Supplementary Tables 12 and 13). Other characteristics are shown in Table 1 and Supplementary Tables 1 and 6–13.

**Table 1:** Descriptive characteristics of cohort participants

	9 years					17 years					24 years				
Variables	Male		Female		*P*-value	Male		Female		*P*-value	Male		Female		*P*-value
	*N*	Mean (SD)	N	Mean (SD)		*N*	Mean (SD)	*N*	Mean (SD)		*N*	Mean (SD)	*N*	Mean (SD)	
*Anthropometry*															
Age (years)	1578	9.84 (0.30)	1898	9.82 (0.29)	0.207	1720	17.72 (0.32)	2143	17.72 (0.34)	0.753	955	24.58 (0.72)	1502	24.50 (0.74)	**0.008**
Height (m)	1567	1.40 (0.06)	1866	1.39 (0.06)	**0.001**	1694	1.79 (0.07)	2112	1.65 (0.06)	**<0.0001**	950	1.80 (0.07)	1484	1.66 (0.06)	**<0.0001**
^a^Weight (kg)	1574	32.80 (7.60)	1897	33.40 (9.0)	**0.026**	1697	69.40 (14.45)	2114	60.40 (13.50)	**<0.0001**	949	77.80 (17.65)	1484	64.65 (17.10)	**<0.0001**
Somatic maturation (*n*,%)	1542	0	1846	20 (0.9)	**<0.0001**	1601	1601 (100)	1927	1927 (100)	NA	NA				
Ethnicity- White (*n*,%)	1566	1498 (95.7)	1925	1848 (96.0)	0.670	NA					NA				
*Body composition*															
^a^Total fat mass (kg)	1508	5.65 (4.93)	1799	8.47 (5.99)	**<0.0001**	1676	10.28 (9.81)	2080	19.24 (10.44)	**<0.0001**	928	17.91 (10.35)	1436	22.12 (11.91)	**<0.0001**
^a^Trunk fat mass (kg)	1508	2.02 (2.01)	1799	3.26 (2.83)	**<0.0001**	1676	5.30 (5.46)	2080	9.46 (5.63)	**<0.0001**	928	8.79 (6.50)	1436	9.91 (6.65)	**<0.0001**
Lean mass (kg)	1508	25.25 (3.75)	1799	23.26 (3.84)	**<0.0001**	1676	54.83 (8.13)	2080	37.68 (5.16)	**<0.0001**	928	56.09 (10.07)	1436	40.82 (6.79)	**<0.0001**
^a^Total fat mass index (kg/m^2^)	1500	2.89 (2.37)	1767	4.37 (2.98)	**<0.0001**	1665	3.18 (3.09)	2072	7.03 (3.67)	**<0.0001**	926	5.57 (3.27)	1434	7.96 (4.33)	**<0.0001**
^a^Trunk fat mass index (kg/m^2^)	1500	1.02 (0.98)	1767	1.70 (1.40)	**<0.0001**	1665	1.64 (1.70)	2072	3.46 (2.00)	**<0.0001**	926	2.72 (2.00)	1434	3.60 (2.37)	**<0.0001**
^a^Lean mass index (kg/m^2^)	1500	12.91 (1.12)	1767	12.06 (1.22)	**<0.0001**	1665	17.11 (1.94)	2072	13.78 (1.52)	**<0.0001**	926	17.29 (2.44)	1434	14.76 (1.96)	**<0.0001**
^a^Body mass index (kg/m^2^)	1567	16.70 (2.78)	1865	17.27 (3.51)	**<0.0001**	1694	21.54 (4.05)	2112	21.95 (4.42)	**<0.0001**	949	23.98 (4.79)	1483	23.51 (5.70)	0.177
*Metabolic profile*															
Total Cholesterol (mmol/L)	1121	4.19 (0.65)	1293	4.32 (0.62)	**<0.0001**	1277	3.56 (0.62)	1310	3.94 (0.69)	**<0.0001**	840	4.34 (0.85)	1174	4.48 (0.82)	**<0.0001**
High density lipoprotein (mmol/L)	1121	1.44 (0.31)	1293	1.38 (0.30)	**<0.0001**	1277	1.19 (0.26)	1310	1.35 (0.32)	**<0.0001**	840	1.40 (0.37)	1174	1.66 (0.42)	**<0.0001**
Low density lipoprotein (mmol/L)	1121	2.24 (0.58)	1293	2.42 (0.56)	**<0.0001**	1277	2.00 (0.56)	1310	2.21 (0.63)	**<0.0001**	838	2.46 (0.79)	1174	2.40 (0.74)	0.071
^a^Triglyceride (mmol/L)	1121	0.98 (0.60)	1293	1.04 (0.60)	**0.005**	1277	0.74 (0.34)	1310	0.76 (0.37)	0.096	839	0.87 (0.55)	1174	0.81 (0.47)	**<0.0001**
Glucose (mmol/L)	NA					1277	5.16 (0.69)	1310	4.91 (0.51)	**<0.0001**	840	5.46 (0.58)	1174	5.22 (0.63)	**<0.0001**
^a^Insulin (mU/L)	1114	8.29 (11.06)	1283	7.98 (10.64)	0.845	1259	6.05 (4.19)	1285	7.33 (4.37)	**<0.0001**	840	6.92 (4.94)	1174	7.75 (5.56)	**<0.0001**
^a^C-reactive protein (mg/L)	1121	0.15 (0.27)	1293	0.26 (0.51)	**<0.0001**	1277	0.44 (0.68)	1310	0.67 (1.28)	**<0.0001**	751	0.63 (1.10)	1100	0.99 (2.13)	**<0.0001**
*Vascular measures*															
Pulse rate (beat/mins)	1554	77 (10)	1872	81 (11)	**<0.0001**	1718	63 (9)	2138	68 (10)	**<0.0001**	953	64 (10)	1495	69 (10)	**<0.0001**
Systolic blood pressure (mm Hg)	1554	102 (9)	1872	103 (9)	**0.013**	1718	120 (9)	2138	110 (8)	**<0.0001**	953	122 (10)	1495	112 (10)	**<0.0001**
Diastolic blood pressure (mm Hg)	1554	57 (6)	1872	58 (6)	**<0.0001**	1718	63 (6)	2138	65 (6)	**<0.0001**	953	67 (8)	1495	66 (8)	**0.004**
^a^Carotid-femoral PWV (m/s)	NA					1720	6.03 (0.70)	2143	5.54 (0.62)	**<0.0001**	641	6.47 (1.21)	1012	5.90 (1.03)	**<0.0001**
^a^Carotid IMT (mm)	NA					1720	0.48 (0.05)	2143	0.47 (0.04)	**<0.0001**	533	0.47 (0.05)^b^	866	0.45 (0.05)^b^	**<0.0001**
*Lifestyle factors*															
Smoked cigarettes in the past 30 days (*n*,%)	NA					1491	369 (21.5)	1853	542 (25.3)	0.004	674	171 (25.4)	1105	284 (25.7)	0.911
MVPA (mins/day)	NA					NA					222	46 (43)	411	42 (35)	**0.030**
Family history of H-D-C-V (*n*,%)	NA					1719	492 (28.6)	2140	670 (31.3)	0.072	NA				

*H-D-C-V* hypertension/diabetes/high cholesterol/vascular disease, *IMT* intima-media thickness, *MVPA* moderate to vigorous physical activity, *NA* not available/applicable, *PWV* pulse wave velocity, *P*-value for sex difference

The values are means (standard deviations) and ^a^median (interquartile range) except for maturation status and lifestyle factors in percentage. Differences between sexes were tested using Student’s *t*-test for normally distributed continuous variables, Mann–Whitney U test for skewed continuous variables, and Chi-square test for dichotomous variable

^b^Mean cIMT measurement at 24 years whereas maximum cIMT measurement for males is 0.54 mm (0.09) and for females is 0.53 mm (0.08)

A 2-sided *P*-value < 0.05 (in bold) is considered statistically significant

### Cross-sectional associations of fat mass, lean mass, and blood pressure with cfPWV and cIMT at age 24 years

At the age of 24 years, lean mass and systolic and diastolic BP were positively associated with cfPWV after multivariable adjustments for cardiometabolic and lifestyle risk factors, but BMI had negative associations with cfPWV after similar adjustments (Table 2). Lean mass, LMI, and systolic BP had positive associations with cIMT, while BMI was negatively associated with cIMT. Participants with very high lean mass and high systolic BP had higher cfPWV and cIMT than those in other categories after similar multivariable adjustment (Supplementary Figure 1). Quartile categories of total fat mass and trunk fat mass at 24 years had no linear trend associations with cfPWV and cIMT at age 24 years (Supplementary Figures 2–4). Most of these results were consistent with the nonimputed regression model findings (Supplementary Table 7).

**Table 2:** Cross-sectional association of fat mass, lean mass and blood pressure with carotid-femoral pulse wave velocity and carotid intima-media thickness at age 24 years

*N* = 1799	Pulse wave velocity (m/s)				Carotid intima-media thickness (mm)			
	Univariate analysis		Multivariable analysis		Univariate analysis		Multivariable analysis	
	*β* (95% CI)	*p*-value	*β* (95% CI)	*p*-value	*β* (95% CI)	*p*-value	*β* (95% CI)	*P*-value
Total fat mass (kg)	0.029 (0.011– 0.046)	**0.001**	−0.007 (−0.030– 0.016)	0.564	0.004 (−0.008–0.015)	0.547	−0.032 (−0.047– −0.018)	**<0.0001**
Trunk fat mass (kg)	0.020 (0.005 – 0.034)	**0.007**	−0.010 (−0.029– 0.008)	0.281	0.001 (−0.009–0.010)	0.978	−0.028 (−0.040– −0.016)	**<0.0001**
Lean mass (kg)	0.160 (0.107– 0.213)	**<0.0001**	0.117 (0.054–0.180)	**<0.0001**	0.132 (0.097–0.166)	**<0.0001**	0.146 (0.103−0.188)	**<0.0001**
Total fat mass index (kg/m^2^)	0.018 (0.0001– 0.035)	**0.045**	−0.016 (−0.038– 0.007)	0.166	0.0001 (−0.012– 0.011)	0.970	−0.030 (−0.044–−0.015)	**<0.0001**
Trunk fat mass index (kg/m^2^)	0.012 (−0.002– 0.027)	0.088	−0.016 (−0.034– 0.002)	0.087	−0.002 (−0.012– 0.007)	0.620	−0.026 (−0.038– −0.014)	**<0.0001**
Lean mass index (kg/m^2^)	0.085 (0.021– 0.149)	**0.009**	−0.006 (−0.082– 0.070)	0.873	0.138 (0.096–0.180)	**<0.0001**	0.146 (0.095–0.196)	**<0.0001**
Body mass index (kg/m^2^)	0.058 (0.018– 0.097)	**0.004**	−0.072 (−0.131– −0.013)	**0.016**	0.040 (0.013–0.066)	**0.003**	−0.060 (−0.099– −0.021)	**0.002**
Systolic blood pressure (mm Hg)	0.001 (0.0001– 0.001)	**<0.0001**	0.001 (0.0001–0.001)	**<0.0001**	0.001 (0.0001– 0.001)	**<0.0001**	0.001 (0.0001–0.001)	**<0.0001**
Diastolic blood pressure (mm Hg)	0.001 (0.001– 0.002)	**<0.0001**	0.001 (0.0001–0.001)	**<0.0001**	0.0001 (−0.0001– 0.001)	0.212	0.0001 (−0.0001– 0.001)	0.284

Univariate analysis was adjusted for sex. Multivariable analysis was adjusted for age, sex, low-density lipoprotein, C-reactive protein, fasting blood glucose, systolic blood pressure and fat mass and/or lean mass depending on the predictor, moderate to vigorous physical activity, smoking status and family history of hypertension/diabetes/high cholesterol/vascular disease. Predictors and outcomes were skewed variables but were logarithmically transformed before linear regression analyses. *β* unstandardized regression coefficient, *CI* confidence interval. Multiple imputations were used to account for missing covariates, all predictors and outcomes had complete cases (*n* = 1799)

A 2-sided *P*-value < 0.05 (in bold) is considered statistically significant

### Cumulative effect of exposure to fat mass, lean mass, and blood pressure from ages 9–24 years on cfPWV and cIMT at 24 years

Accumulated high exposures to total fat mass, BMI, lean mass, systolic BP, and diastolic BP from childhood through young adulthood were associated with faster cfPWV at age 24 years after adjustment for cardiometabolic and lifestyle risk factors (Table 3). Cumulative high exposures to lean mass, LMI, BMI and systolic BP were strongly associated with higher cIMT at age 24 years.

**Table 3:** Cumulative effect of exposure to fat mass, lean mass, and blood pressure from age 9 to 24 years on carotid-femoral pulse wave velocity and carotid intima media thickness at 24 years of age

*N* = 1852	Carotid-femoral pulse wave velocity				Carotid intima-media thickness			
	Model 1		Model 2		Model 1		Model 2	
	Effect estimate (95% CI)	*p*-value	Effect estimate (95% CI)	*p*-value	Effect estimate (95% CI)	*p*-value	Effect estimate (95% CI)	*p*-value
*Total fat mass (kg)*								
Low category	Reference	–	Reference	–	Reference	–	Reference	–
Moderate category	0.007 (0.002–0.012)	0.849	−0.001 (−0.006–0.004)	0.763	0.002 (−0.002−0.006)	0.923	−0.0003 (−0.004−0.003)	0.883
High category	−0.001 (−0.005–−0.004)	**0.007**	0.006 (0.001–0.011)	**0.020**	−0.0002 (−0.004–0.003)	0.316	0.002 (−0.002–0.008)	0.347
*Trunk fat mass (kg)*								
Low category	Reference	–	Reference	–	Reference	–	Reference	–
Moderate category	0.001 (−0.004–0.006)	0.656	0.001 (−0.004–0.006)	0.745	−0.001 (−0.005–0.002)	0.503	−0.001 (−0.005–0.002)	0.472
High category	0.006 (0.001–0.011)	**0.024**	0.005 (−0.0001–0.010)	0.053	0.001 (−0.003–0.004)	0.751	0.001 (−0.003–0.004)	0.736
*Lean mass (kg)*								
*Low category*	Reference	–	Reference	–	Reference	–	Reference	–
Moderate category	0.003 (−0.002− 0.008)	0.209	0.003 (−0.002−0.008)	0.309	0.006 (0.003 – 0.009)	**<0.0001**	0.006 (0.003−0.009)	**<0.0001**
High category	0.015 (0.009−0.021)	**<0.0001**	0.014 (0.008−0.020)	**<0.0001**	0.014 (0.010−0.018)	**<0.0001**	0.014 (0.010−0.017)	**<0.0001**
*Total fat mass index (kg/m*^*2*^*)*								
Low category	Reference	–	Reference	–	Reference	–	Reference	–
Moderate category	−0.002 (−0.007–0.003)	0.514	−0.002 (−0.007–0.003)	0.486	−0.002 (−0.005–0.002)	0.341	−0.002 (−0.005–0.002)	0.329
High category	0.003 (−0.002–0.009)	0.245	0.003 (−0.003–0.008)	0.349	0.001 (−0.003–0.005)	0.591	0.001 (−0.003–0.005)	0.581
*Trunk fat mass index (kg/m*^*2*^*)*								
Low category	Reference	–	Reference	–	Reference	–	Reference	–
Moderate category	−0.002 (−0.007− 0.003)	0.411	−0.002 (−0.007–0.003)	0.400	–0.003 (−0.007–0.001)	0.126	−0.003 (−0.007–0.001)	0.112
High category	0.002 (−0.003− 0.007)	0.455	0.001 (–0.004–0.007)	0.588	−0.0001 (−0.004–0.004)	0.956	−0.0001 (−0.004–0.004)	0.975
*Lean mass index (kg/m*^*2*^*)*								
Low category	Reference	–	Reference	–	Reference	–	Reference	–
Moderate category	−0.006 (−0.011–−0.002)	**0.010**	−0.006 (−0.011–−0.002)	**0.008**	0.003 (−0.004–0.006)	0.083	0.003 (−0.004–0.006)	0.083
High category	0.0001 (−0.005–0.005)	0.977	−0.006 (−0.006–0.005)	0.934	0.010 (0.007–0.014)	**<0.0001**	0.010 (0.007–0.014)	**<0.0001**
*Body mass index (kg/m*^*2*^*)*								
Low category	Reference	–	Reference	–	Reference	–	Reference	–
Moderate category	0.003 (−0.002–0.008)	0.194	0.003 (−0.002–0.007)	0.259	0.001 (−0.002–0.004)	0.726	0.001 (−0.003–0.004)	0.770
High category	0.008 (0.003–0.013)	**0.001**	0.007 (0.002–0.012)	**0.004**	0.007 (0.004–0.011)	**<0.0001**	0.007 (0.004–0.011)	**<0.0001**
*Systolic blood pressure (mm Hg)*								
Low category	Reference	–	Reference	–	Reference	–	Reference	–
Moderate category	0.005 (0.001 – 0.010)	**0.022**	0.005 (0.001 – 0.009)	**0.033**	0.002 (-0.001–0.005)	0.101	0.002 (−0.001–0.005)	0.105
High category	0.013 (0.008–0.017)	**<0.0001**	0.011 (0.006–0.016)	**<0.0001**	0.012 (0.009–0.015)	**<0.0001**	0.012 (0.009–0.015)	**<0.0001**
*Diastolic blood pressure (mm Hg)*								
Low category	Reference	**–**	Reference	**–**	Reference	**–**	Reference	**–**
Moderate category	0.008 (0.003–0.012)	**0.001**	0.008 (0.003–0.012)	**0.001**	0.001 (−0.002–0.004)	0.463	0.001 (−0.002–0.004)	0.458
High category	0.016 (0.012–0.021)	**<0.0001**	0.016 (0.011–0.020)	**<0.0001**	0.002 (−0.001–0.005)	0.239	0.002 (−0.001–0.005)	0.286

Effect estimates and CI, confidence interval, from linear mixed model analyses. Differences and associations with *p*-value < 0.05 are considered statistically significant

Model 1 was adjusted for age at baseline, sex, time, systolic blood pressure, low-density lipoprotein, C-reactive protein, fasting blood glucose, and fat mass and/or lean mass depending on the predictor

Model 2 Further adjustment of model 1 for moderate to vigorous physical activity at 24 years, smoking status, and family history of hypertension/diabetes/high cholesterol/vascular disease

A 2-sided *P*-value < 0.05 (in bold) is considered statistically significant

### Cumulative effect of exposure to fat mass, lean mass, and blood pressure from ages 9–24 years on a 7-year change in cfPWV and cIMT from ages 17–24 years

Accumulated high exposures to lean mass, systolic BP, and diastolic BP from ages 9–24 years were positively associated with the 7-year increase in cfPWV after adjustment for cardiometabolic and lifestyle risk factors (Table 4). There were sex differences in the associations of cumulative high exposures to lean mass with the 7-year increase in cfPWV. We found a direct association among males (0.025 m/s [(0.015 to 0.035); *p* < 0.0001]), but an inverse association was seen among females (−0.007 m/s [−0.013 to −0.001); *p* = 0.017]). Exposure to BMI, total fat mass, trunk fat mass, total FMI, and trunk FMI at ages 9–24 years did not have any effect on the increase in cfPWV. Further adjustments for insulin concentration, diet, socioeconomic status, or ethnicity did not alter the results (data not shown). Among normal-weight participants (BMI < 24.99 kg/m^2^), cumulative high exposures to lean mass but not total or trunk fat mass were directly associated with the 7-year increase in cfPWV. However, among participants with overweight and obesity, neither lean mass, total fat mass, trunk fat mass nor BMI was associated with cfPWV progression (Supplementary Table 14). Among normal-weight males, cumulative high lean mass exposure was directly associated with cfPWV progression (0.027 m/s [(0.017 to 0.037); *p* < 0.0001]) but was inversely associated among normal-weight females (−0.009 m/s [(−0.018 – −0.0001); *p* = 0.048]). Among overweight and obese males and females, cumulative high lean mass was not associated with cfPWV progression. Cumulative high exposure to systolic BP was directly associated with the 7-year change in cfPWV among participants with overweight and obesity but not among normal-weight individuals (Supplementary Table 14). The interaction effect between cumulative high exposure to systolic BP and cumulative lean mass exposure in relation to the 7-year increase in cfPWV was 0.079 m/s [(CI 0.062 to 0.097), *P* < 0.0001].

**Table 4:** Cumulative effect of exposure to fat mass, lean mass, and blood pressure from age 9 to 24 years on a 7-year change in carotid-femoral pulse wave velocity and carotid intima-media thickness from age 17 to 24 years

*N* = 3863	Carotid femoral pulse wave velocity				Carotid intima-media thickness			
	Model 1		Model 2		Model 1		Model 2	
	Effect estimate (95% CI)	*p*-value	Effect estimate (95% CI)	*p*-value	Effect estimate (95% CI)	*p*-value	Effect estimate (95% CI)	*p*-value
*Total fat mass (kg)*								
Low category	Reference	–	Reference	–	Reference	–	Reference	–
Moderate category	0.0002 (−0.004–0.005)	0.898	0.0003 (−0.004–0.005)	0.872	0.001 (−0.003–0.005)	0.517	0.001 (−0.003–0.005)	0.557
High category	0.001 (−0.003–0.006)	0.588	0.001 (−0.004–0.006)	0.627	0.003 (−0.001–0.006)	0.110	0.003 (−0.001–0.006)	0.108
*Trunk fat mass (kg)*								
Low category	Reference	–	Reference	–	Reference	–	Reference	–
Moderate category	0.0004 (−0.005–0.006)	0.354	0.0003 (−0.005–0.005)	0.875	0.0003 (−0.003–0.004)	0.872	0.0003 (−0.003–0.004)	0.863
High category	0.001 (−0.004–0.006)	0.612	0.001 (−0.004–0.006)	0.667	0.002 (−0.003–0.006)	0.444	0.002 (−0.002–0.006)	0.385
*Lean mass (kg)*								
Low category	Reference	–	Reference	–	Reference	–	Reference	–
Moderate category	−0.0001 (−0.004–0.004)	0.944	0.0001 (−0.004–0.004)	0.996	0.007 (0.004–0.010)	**<0.0001**	0.007 (0.003–0.010)	**<0.0001**
High category	0.005 (0.001–0.009)	**0.027**	0.006 (0.001–0.010)	**0.022**	0.012 (0.007–0.016)	**<0.0001**	0.012 (0.008–0.016)	**<0.0001**
*Total fat mass index (kg/m*^*2*^*)*								
Low category	Reference	–	Reference	–	Reference	–	Reference	–
Moderate category	0.003 (−0.003–0.008)	0.348	0.003 (−0.003–0.008)	0.355	−0.0002 (−0.004–0.003)	0.913	−0.0002 (−0.004–0.004)	0.919
High category	0.001 (−0.006–0.008)	0.779	0.001 (−0.007–0.008)	0.833	0.001 (−0.003–0.005)	0.634	−0.001 (−0.003–0.006)	0.617
*Trunk fat mass index (kg/m*^*2*^*)*								
Low category	Reference	–	Reference	–	Reference	–	Reference	–
Moderate category	−0.0002 (−0.005–0.005)	0.932	−0.0003 (−0.005–0.005)	0.926	−0.0002 (−0.004–0.004)	0.990	−0.0005 (−0.004–0.004)	0.672
High category	−0.0001 (−0.006–0.006)	0.978	−0.0002 (−0.006–0.0006)	0.993	0.001 (−0.003–0.005)	0.677	0.001 (−0.003–0.004)	0.649
*Lean mass index (kg/m*^*2*^*)*								
Low category	Reference	–	Reference	–	Reference	–	Reference	–
Moderate category	−0.003 (−0.007–0.002)	0.249	−0.002 (−0.007–0.002)	0.312	0.004 (0.001–0.007)	**0.019**	0.004 (0.001–0.007)	**0.025**
High category	0.004 (−0.007–0.009)	0.087	0.005 (−0.005–0.010)	0.081	0.006 (0.003–0.010)	**0.001**	0.006 (0.003–0.010)	**0.001**
*Body mass index (kg/m*^*2*^*)*								
Low category	Reference	–	Reference	–	Reference	–	Reference	–
Moderate category	0.002 (−0.002–0.005)	0.356	0.002 (−0.002–0.006)	0.306	0.003 (−0.001–0.006)	0.112	0.003 (−0.001–0.006)	0.120
High category	−0.001 (−0.005–0.004)	0.719	−0.001 (−0.005–0.004)	0.727	0.007 (0.003–0.011)	**0.001**	0.007 (0.003–0.011)	**0.001**
*Systolic blood pressure (mm Hg)*								
Low category	Reference	–	Reference	–	Reference	–	Reference	–
Moderate category	0.007 (0.003–0.012)	**0.003**	0.007 (0.003–0.012)	**0.003**	0.001 (−0.002–0.004)	0.538	0.001 (−0.003–0.004)	0.526
High category	0.013 (0.009–0.017)	**<0.0001**	0.013 (0.009–0.017)	**<0.0001**	0.009 (0.006–0.014)	**<0.0001**	0.010 (0.006–0.014)	**<0.0001**
*Diastolic blood pressure (mm Hg)*								
Low category	Reference	**–**	Reference	**–**	Reference	**–**	Reference	**–**
Moderate category	0.015 (0.011–0.019)	**<0.0001**	0.015 (0.011–0.018)	**<0.0001**	−0.0002 (−0.003–0.003)	0.890	−0.0002 (−0.003–0.003)	0.891
High category	0.023 (0.020–0.027)	**<0.0001**	0.023 (0.019–0.027)	**<0.0001**	0.001 (−0.002–0.004)	0.647	0.001 (−0.002–0.004)	0.622

Effect estimates and CI, confidence interval, from linear mixed model analyses. Differences and associations with *p*-value < 0.05 are considered statistically significant

Model 1 was adjusted for age at baseline, sex, time, systolic blood pressure, low-density lipoprotein, C-reactive protein, fasting blood glucose, and fat mass and/or lean mass depending on the predictor

Model 2 Further adjustment of model 1 for moderate to vigorous physical activity at 24 years, smoking status, and family history of hypertension/diabetes/high cholesterol/vascular disease

A 2-sided *P*-value < 0.05 (in bold) is considered statistically significant

The combined high exposure to lean mass, LMI, BMI, and systolic BP from ages 9–24 years was positively associated with an increase in cIMT from ages 17–24 years after full adjustments. We found no association of exposures to diastolic BP, total fat mass, trunk fat mass, total FMI, or trunk FMI from ages 9–24 years with the 7-year increase in cIMT. Most of these results were consistent with the complete case analysis (Supplementary Table 9), and there were no sex differences. Among normal-weight participants (BMI < 24.99 kg/m^2^), cumulative high exposure to lean mass was directly associated, while total fat mass and trunk fat mass were inversely associated, with the 7-year increase in cIMT (Supplementary Table 14). However, among overweight and obese participants, lean mass, total fat mass, trunk fat mass, and BMI lacked any association with cIMT progression. Cumulative high exposure to systolic BP was directly associated with the 7-year change in cIMT among normal-weight participants but not among individuals with overweight and obesity (Supplementary Table 14).

Among participants with cIMT in the ≥90th percentile, indicating subclinical atherosclerosis, neither total fat mass, lean mass, nor systolic BP was associated with cfPWV progression. However, among participants with cfPWV in the ≥90th percentile, indicating vascular organ damage, only systolic BP but not total fat mass or lean mass was associated with cIMT progression (Table 5).

**Table 5:** Role of the risk of vascular organ damage (subclinical atherosclerosis and arteriosclerosis) on the cumulative effect of total fat mass, lean mass, and systolic blood pressure from 9–24 years on 7-year change in carotid-femoral pulse wave velocity and carotid intima-media thickness from ages 17 to 24 years

*N* = *3863*	cfPWV progression (17–24 years)			
	Participants with <90th percentile cIMT (normal cIMT)		Participants with ≥90th percentile cIMT (atherosclerosis)	
	Effect estimate (95% CI)	*p-*value	Effect estimate (95% CI)	*p*-value
*Cumulative Systolic blood pressure (mmHg) (*9–24 *years)*				
Low category	Reference	–	Reference	–
Moderate category	0.004 (0.0004–0.007)	**0.028**	0.005 (−0.014–0.023)	0.608
High category	0.007 (0.003–0.011)	**0.001**	0.013 (−0.003–0.028)	0.102
*Cumulative Lean mass (kg) (*9–24 *years)*				
Low category	Reference	–	Reference	–
Moderate category	0.001 (−0.003–0.005)	0.698	0.001 (−0.014–0.016)	0.903
High category	0.008 (0.002–0.014)	**0.009**	0.012 (−0.005–0.029)	0.152
*Cumulative Total fat mass (kg) (*9–24 *years)*				
Low category	Reference	–	Reference	–
Moderate category	0.002 (−0.002–0.006)	0.229	0.002 (−0.009–0.014)	0.694
High category	0.003 (−0.002–0.009)	0.228	−0.003 (−0.020–0.014)	0.755

*N* = *3863*	cIMT progression (17–24 years)			
	Participants with < 90th percentile cfPWV (Optimal stiffness)		Participants with ≥ 90th percentile cfPWV (arteriosclerosis)	
	Effect estimate (95% CI)	*p*-value	Effect estimate (95% CI)	*p-*value
*Cumulative Systolic blood pressure (mmHg) (9–24 years)*				
Low category	Reference	–	Reference	–
Moderate category	0.001 (−0.002–0.004)	0.523	0.008 (−0.003–0.020)	0.155
High category	0.008 (0.005–0.011)	**<0.0001**	0.013 (0.001–0.025)	**0.035**
*Cumulative Lean mass (kg)* (9–24 *years)*				
Low category	Reference	–	Reference	–
Moderate category	0.005 (0.002–0.008)	**<0.0001**	0.0001 (−0.013–0.013)	0.987
High category	0.010 (0.005 – 0.013)	**<0.0001**	0.002 (−0.012–0.017)	0.738
*Cumulative Total fat mass (kg) (9–24 years)*				
Low category	Reference	**–**	Reference	–
Moderate category	−0.001 (−0.004–0.003)	0.697	0.009 (−0.003–0.020)	0.130
High category	0.002 (−0.001–0.006)	0.214	0.001 (−0.012–0.013)	0.895

Model was adjusted for age at baseline, sex, time in years from baseline through follow-up, low-density lipoprotein, C-reactive protein, fasting blood glucose, moderate to vigorous physical activity at 24 years, smoking status, and family history of hypertension/diabetes/high cholesterol/vascular disease, in addition to fat mass, systolic blood pressure, and/or lean mass depending on the predictor

A 2-sided *P*-value < 0.05 (in bold) is considered statistically significant

## Discussion

In a population-based cohort, we found that changes in cfPWV and cIMT from adolescence through young adulthood likely reflect arterial structural adaptation to an increased lean mass and systolic BP from childhood, independent of cardiometabolic and lifestyle risk factors and vascular organ damage. We established this reasoning from the following observations. First, we found that higher lean mass and systolic BP were cross-sectionally associated with an increased cfPWV and cIMT at 24 years, while fat mass and BMI were inversely associated with cIMT at age 24 years after controlling for lean mass. Second, we found that accumulated exposures to high lean mass and systolic BP from ages 9–24 years independently and strongly predicted faster cfPWV and higher cIMT at age 24 years. Last, we observed that the highest cumulative exposures to lean mass and systolic BP from childhood to young adulthood were independently associated with the 7-year increase in cfPWV and cIMT from adolescence through young adulthood. Our findings suggest that higher BMI, a surrogate measure of adiposity that has been strongly linked with increased arterial stiffness [2] and intima-media thickness [11, 12, 26], in a normal young population may be unrelated to the adverse effect on body fat but rather to a physiologic vascular wall remodeling process from increased lean mass and systolic BP [7].

### The effect of cumulative exposures to fat mass, lean mass, and blood pressure on cfPWV

Arterial stiffness, assessed by cfPWV, is a strong predictor of future cardiovascular events and all-cause mortality in adults [5]. However, since clinical cardiovascular events do not typically occur in childhood and adolescence, cfPWV is used as a surrogate risk marker for atherosclerotic cardiovascular disease [2–4]. It is well established that childhood and adolescent obesity is associated with an increased risk of central arterial stiffness measured between the carotid-femoral segment [2, 3, 5]. Nonetheless, there is limited evidence on the influence of body fat during the long preclinical phase of the arterial disease [3]. A recent study showed that higher fat mass assessed by DEXA and serially measured from ages 9–17 years was associated with an increased cfPWV at age 17 years [3]. This finding is congruent with the results of our present study, conducted with the same cohort, where a serially measured higher body fat from ages 9–24 years was associated with a faster cfPWV at 24 years, i.e., 7 years after the previous study’s outcome timepoint. Nevertheless, we observed a weaker effect of cumulative exposure to higher fat mass on cfPWV in young adulthood than in adolescence [3]. Further investigation revealed that accumulated fat mass from childhood to young adulthood did not have an effect on the 7-year increase in cfPWV between the ages of 17 and 24 years. It is known that arterial compliance increases with age since arterial diameter, function, and structure increase during the course of childhood growth and development [14]. This physiological increase may temper an age-associated increase in arterial stiffness and the deleterious effect of fat on cfPWV in a young population [14].

Regarding the pattern of fat distribution, we found that cumulative exposure to trunk fat, a measure of visceral fat, was not related to the 7-year increase in cfPWV. However, there was a borderline association between cumulative exposure to trunk fat and cfPWV at 24 years after multivariate adjustments. Evidence supports a stronger relationship between visceral fat and cardiometabolic risk factors, such as dyslipidemia, elevated systolic BP, and insulin resistance, rather than altered arterial structure and function [3, 27]. Cumulative exposure to high BMI from ages 9–24 years was associated with faster cfPWV at 24 years of age, similar to the findings of a recall-by-genotype report in the same cohort at the age of 17 years [8]. However, our cross-sectional findings at age 24 years revealed that BMI was associated with slower cfPWV, a protective factor, akin to multivariable regression results at 17 years [8]. A Mendelian randomization analysis in the same cohort reported that BMI was unrelated to cfPWV at 17 years [8]. These conflicting results may reflect growth- and maturation-induced changes in BMI on cfPWV [8]. Altogether, our results reinforce previous knowledge that the association between BMI and body fat is weak and its use in predicting arterial disease risk in childhood and adolescence is poor [3, 13]. In addition, the association between cumulative exposure to high BMI and cfPWV in young adulthood may be due to lean mass and not body fat per se.

Persistent exposure to high lean mass from childhood to young adulthood had very strong associations with faster cfPWV at age 24 years and with the 7-year increase in cfPWV from ages 17–24 years. Studies in adults have associated lower lean mass, independent of body fat, with increased cardiovascular- and all-cause mortality risk [28–30]. Our findings of a 15-year increase in lean mass from childhood to young adulthood, independent of cardiometabolic and lifestyle risk factors, may therefore suggest a central artery protective effect, which may increase the healthy lifespan, plausibly because increased lean mass, i.e., higher skeletal and cardiac muscle mass, is related to higher stroke volume and cardiac output via adaptation of the cardiovascular system [31]. We observed that among normal-weight males, cumulative high and moderate lean mass exposures were strongly associated with a greater increase in cfPWV. However, among normal-weight females, cumulative high lean mass exposure was inversely associated with an increase in cfPWV. There was no significant association between cumulative lean mass and cfPWV progression among overweight and obese males and females. The disparity in the lean mass relationship with cfPWV progression among males and females may be due to the significantly lower lean mass to fat mass ratio in females, where males had an average of 17 kg more lean mass than females at ages 17 and 24 years. In addition, a higher fat mass and hormonal influence may contribute to vasodilation in females despite a steadily increasing lean mass from ages 9 through 24 years.

Thus, in apparently young adults, particularly among normal-weight males, a higher lean mass in association with a higher cfPWV, albeit within the physiologic range, may enhance cardiovascular health in contrast to the deleterious effect of a lower lean mass and an elevated cfPWV in the elderly population [32]. It is known that a rapid decline in muscle mass and BP elevation occurs with aging [15, 32], so a disruption of this adaptive mechanism may result in the deleterious effect of lower lean mass in association with increased cfPWV. Efforts targeted at improving lower lean mass to fat mass ratio from adolescence, especially among females, may yield clinical and public health benefits in mitigating the adverse effect of elevated cfPWV in later life. The separate associations of 9 to 24-year cumulative lean mass and LMI with cfPWV at 24 years, the 7-year increase in cfPWV, or the cross-sectional associations showed contrasting results. This is due to lean mass being indexed for height. In our study population, height had a near-perfect correlation with lean mass, and cfPWV was height dependent. Thus, the negative associations between cumulative LMI and cfPWV at 24 years or the lack of associations between cumulative LMI and the 7-year increase in cfPWV may be due to the confounding role of body height. Therefore, analyses with body height and lean mass in the same model or LMI in relation to cfPWV may produce spurious results, which should be cautiously interpreted.

Persistent exposure to high systolic or diastolic BP from ages 9–24 years was strongly and independently associated with high cfPWV at the age of 24 years and the 7-year increase in cfPWV after controlling for total fat mass, lean mass, metabolic factors, and lifestyle risk factors. An increased BP likely maintains arterial patency and elasticity (vascular adaptation) [4, 10] in response to a higher stroke volume, cardiac output, and muscle proliferation. A statistically significant interaction effect observed between lean mass and BP in relation to cfPWV appears multiplicative in comparison to the independent relationships of lean mass or BP with cfPWV progression. Additionally, cumulative lean mass from 9–24 years contributed a ~25% additional effect to the association between cumulative high exposure to systolic BP from 9–24 years and the increase in cfPWV from 17–24 years. Hence, our results suggest that childhood lean mass and BP may be the primary drivers of central arterial function from adolescence to young adulthood. On the other hand, participants included in the high BP categories had >120 mmHg systolic BP at the age of 24 years, which is reflective of elevated BP [9]. An American Heart Association scientific statement recommended that future studies evaluate the natural history of cfPWV and BP vis-à-vis the rate at which cfPWV and BP increase with age [33]. We found that a 10 mmHg increase in systolic BP from ages 9–24 years was independently associated with a 0.1 m/s increase in cfPWV from ages 17–24 years. An important clinical implication of these findings is that a 1 m/s increase in cfPWV corresponds to an ~15% increase in cardiovascular disease mortality and events in middle-aged and elderly populations [5]. Additionally, elevated cfPWV in adolescence may temporally cause hypertension, obesity, and insulin resistance in young adulthood [20, 21]. Thus, decreasing BP from childhood, especially among overweight and obese individuals, may reduce arterial stiffness and plausibly cardiovascular disease mortality and events later in life [5, 33]. Consistent with the findings described above, lean mass may be a novel confounder in the relationships between BP and cfPWV due to the multiplicative and additive effect of lean mass on BP-vascular stiffness relationship.

### The effect of cumulative exposures to fat mass, lean mass, and blood pressure on cIMT

Evidence on the use of cIMT as a surrogate marker of cardiovascular events in the general population is conflicting [1, 7, 20, 21, 34] because cIMT progression in the elderly reflects atherosclerotic changes [1], whereas in adolescence, cIMT may represent structural remodeling of the arteries [1, 7, 20, 21]. It is pertinent to note that accumulated exposure to all DEXA-measured body fat content (i.e., total fat mass and trunk fat mass) was unrelated to cIMT either at age 24 years or the 7-year change from 17–24 years. However, total fat mass was inversely associated with cIMT at 24 years in the cross-sectional analysis only after controlling for lean mass. Moreover, from 9–24 years, exposure to high BMI, a surrogate measure of obesity, was associated with high cIMT at either time point, which was in agreement with previous reports [11, 12, 26]. We provide a clearer understanding of this phenomenon in that our findings and a Mendelian randomization study suggest that body fat and BMI, respectively, may have no causal role in cIMT in an apparently healthy population [8, 20]. Since BMI poorly discriminates lean mass from body fat [13], previous studies might have unwittingly interpreted the direct association of childhood-through-young adulthood BMI with cIMT in young adulthood as an adverse effect of obesity [11, 12, 26]. None of the measures of adiposity were associated with cIMT in participants with overweight or obesity. However, we observed that cumulative high exposure to total fat mass and trunk fat mass but not BMI was inversely associated with the 7-year cIMT progression in normal-weight young adults. These paradoxical results, independent of metabolic, lipid, and inflammatory profiles, suggest a vascular protective effect of subcutaneous and visceral fat in normal-weight individuals, i.e., the “fat mass paradox”. Therefore, decreasing overweight and obesity in young adulthood, especially from 17–24 years of age, may delay or prevent the progression of subclinical arterial diseases [11, 14, 20]. Our prospective findings confirm and expand the evidence from the Atherosclerosis Risk in Young Adults’ study where weight reduction, estimated with BMI, from adolescence was inversely associated with cIMT at age 28 years [11]. Further research is required to ascertain the mechanism by which fat mass independently attenuates cIMT progression in normal-weight adolescents and young adults [14].

An accumulation of lean mass reflects a cycle of increased skeletal and cardiac muscle mass, higher metabolic demand, elevated stroke volume, elevated systolic BP, and increased cardiac output [14, 27, 31, 32]. Thickening from an increased lean mass occurs in the media layer of the carotid vessel in the young population [7]. We found that cumulative exposure to high systolic BP from childhood to young adulthood was associated with higher cIMT at 24 years and 7-year cIMT progression, independent of cumulative exposure to lean mass, fat mass, metabolic, and lifestyle risk factors. Our result is congruent with a prospective study where trajectories of systolic BP from ages 8–15 years at baseline were strongly associated with higher cIMT measured once after 23 years [10].

Arterial stiffness and cIMT progression have different and overlapping mechanisms in vascular aging processes, while the former may be related to elastin loss and fragmentation, and the latter relates to atherosclerosis [1, 5, 33, 34]. Evidence on the role of BP in these processes has been well studied, but the role of body composition remains unclear [20, 33, 34]. We observed that among participants who were classified in the subclinical atherosclerosis category, the relationship of cumulative lean mass, total fat mass, and BP with arterial stiffness progression was not statistically significant. Subclinical atherosclerosis may have no role in the associations of cumulative high lean mass, fat mass, or BP with arterial stiffness progression in our study population. However, cumulative high systolic BP but not lean mass or total fat mass was associated with progressive carotid wall thickening among participants at risk of arteriosclerosis. This suggests that the BP relationship with cIMT progression may be influenced by vascular aging (elastin loss and fragmentation). Early vascular aging may precede deleterious cumulative high BP exposure, which could be clinically relevant to cIMT progression [20]. Overall, cIMT progression from ages 17–24 years in a young adult population may reflect structural adaptation – intima-media-lean mass deposits – rather than subclinical signs of atherosclerotic diseases enhanced by obesity (BMI).

### Strengths and limitations

We report cross-sectional and longitudinal findings from one of the world’s most extensive prospective birth cohorts (ALSPAC) with minimal undetected bias due to an array of available cardiometabolic and lifestyle factors. The ability to discriminate the effects of lean mass and fat mass components of BMI emphasizes the need for a DEXA-derived measure of body composition when evaluating the association of adiposity with surrogate markers of arterial diseases in the young population. The observational design of this study may have led to residual bias, such as different equipment used in measuring predictors, for instance, automated BP measures, cIMT outcomes, and covariates across the different time points – ages 9, 17, and 24 years. We may not extrapolate our results to other ethnic groups since almost all participants were Caucasian; thus, future studies may investigate this hypothesis among participants from other ethnicities. The prospective design of this study does not allow for the determination of causality; however, our results are enhanced by a Mendelian randomization analysis from the same cohort that reported no causal role between BMI and cfPWV or cIMT at age 17 years [8]. We could not fully control for diet across all time points because dietary variables were available only at the 9- and 15-year clinic visits. However, controlling for diet did not alter the present results. Similarly, physical activity variables objectively measured by an accelerometer at ages 9 and 17 years were unavailable, so we used the measure at 24 years in our analyses. The lack of some lifestyle factor variables at specific time points might have introduced residual bias.

## Conclusion

Accumulated exposures to high total fat mass and trunk fat mass from ages 9–24 years were not associated with 7-year cfPWV or cIMT progression from ages 17–24 years in a young growing population. Our findings suggest that cumulative high exposure to lean mass in association with an increase in cfPWV and cIMT may be reflective of physiological remodeling rather than markers of preclinical arteriosclerotic and atherosclerotic diseases. Thus, the direct associations of early-life BMI exposure, a surrogate measure of obesity, with elevated cfPWV and cIMT in a young apparently healthy population should be cautiously interpreted. Lean mass and BP complementarily drive the increase in cfPWV and cIMT. Sex differences observed in the cumulative associations of lean mass with cfPWV may be due to a consistently higher quantity of lean mass and lean mass to fat mass ratio in males than females.

## Supplementary information

Supplementary information

## Data Availability

Informed consent obtained from ALSPAC participants does not allow the data to be made freely available through any third-party maintained public repository. However, data used for this submission can be made available on request to the ALSPAC Executive. The ALSPAC data management plan describes in detail the policy regarding data sharing, which is through a system of managed open access. Full instructions for applying for data access can be found here: http://www.bristol.ac.uk/alspac/researchers/access/. The ALSPAC study website contains details of all the data that are available (http://www.bristol.ac.uk/alspac/researchers/our-data/).
