# Supplementary material for: Cumulative muscle mass and blood pressure but not fat mass drives arterial stiffness and carotid intima-media thickness progression in the young population and is unrelated to vascular organ damage
Source: Hypertens Res. 2022 Oct 14;46(4):984–99. doi: 10.1038/s41440-022-01065-1 (PMC10073015; doi:10.1038/s41440-022-01065-1)
Supplement: Supplementary file 1 — Supplementary information [file 41440_2022_1065_MOESM1_ESM.pdf]

## DATA SUPPLEMENT

# Cumulative muscle mass and blood pressure but not fat mass drives arterial stiffness and carotid intima-media thickness progression in the young: unrelated to vascular organ damage

Andrew O. Agbaje MD, MPH[1], Alan R. Barker PhD[2], Tomi-Pekka Tuomainen MD, PhD[1]

*[1]Institute of Public Health and Clinical Nutrition, School of Medicine, University of Eastern Finland, Kuopio, Finland. [2]Children's Health and Exercise Research Centre, Sport and Health Sciences, University of Exeter, Exeter, United Kingdom.*

**Content list:**

Expanded material and methods

References

Supplementary Table 1 Characteristics of participants excluded from the study

Supplementary Table 2 Missing data at 24 years of age

Supplementary Table 3 Missing data at 17 years of age

Supplementary Table 4 Variables used in multivariable multiple imputation model at 24 years

Supplementary Table 5 Variables used in multivariable multiple imputation model at 17 years

Supplementary Table 6 Characteristics of cohort participants at age 24 years (imputed data)

Supplementary Table 7 Cross-sectional association of fat mass, lean mass and systolic blood pressure with carotid-femoral pulse wave velocity and carotid intima-media thickness at age 24 years. (Non-imputed data, pairwise deletion)

Supplementary Table 8 Cumulative effect of exposure to fat mass, lean mass, and systolic blood pressure from 9 to 24 years on carotid-femoral pulse wave velocity and carotid intima-media thickness at age 24 years (Non-imputed dataset, listwise deletion)

Supplementary Table 9 Cumulative effect of exposure to fat mass, lean mass, and systolic blood pressure from age 9 to 24 years on a 7-year change in carotid-femoral pulse wave velocity and carotid intima-media thickness from age 17 to 24 years (Non-imputed dataset, listwise deletion)

Supplementary Table 10 Descriptive characteristics of participants based on body mass index classification of normal weight and overweight and obesity at age 17 years

Supplementary Table 11 Descriptive characteristics of participants based on body mass index classification of normal weight and overweight and obesity at age 24 years

Supplementary Table 12 Descriptive characteristics based on systolic blood pressure classification as normotensive or elevated blood pressure and hypertension at age 17 years

Supplementary Table 13 Descriptive characteristics based on systolic blood pressure classification as normotensive or elevated blood pressure and hypertension at age 24 years

Supplementary Table 14 Cumulative effect of exposure to fat mass, lean mass, and systolic blood pressure from age 9 to 24 years on a 7-year change in carotid-femoral pulse wave velocity and carotid intima-media thickness from age 17 to 24 years based on body mass index status at 24 years of normal weight and overweight and obese

Supplementary Figure 1 Inter-quartile differences in the cross-sectional associations of categories of fat mass, lean mass, and blood pressure with carotid-femoral pulse wave velocity and carotid intima-media thickness at age 24 years.

Supplementary Figure 2 Linear trend in the cross-sectional associations of categories of total fat mass, trunk fat mass, and lean mass with carotid-femoral pulse wave velocity at age 24 years.

Supplementary Figure 3 Linear trend in the cross-sectional associations of categories of trunk fat mass, total fat mass, and lean mass with carotid intima-media thickness at age 24 years.

Supplementary Figure 4 Linear trend in the cross-sectional associations of categories of body mass index, systolic and diastolic blood pressure with carotid-femoral pulse wave velocity and carotid intima-media thickness at age 24 years.

## Expanded Material and Methods

### Data Availability Statement

The informed consent obtained from the Avon longitudinal study of parents and children (ALSPAC) participants does not allow the data to be made freely available through any third-party maintained public repository. However, data used for this submission can be made available on request to the ALSPAC Executive. The ALSPAC data management plan describes in detail the policy regarding data sharing, which is through a system of managed open access. Full instructions for applying for data access can be found here: http://www.bristol.ac.uk/alspac/researchers/access/.

### Study cohort

Data were from the ALSPAC birth cohort, which investigates factors that influence normal childhood development and growth. Altogether, 14,541 pregnant women from Avon, southwestern England, UK, who had a total of 14,676 fetuses, were enrolled between April 1, 1991, and December 31, 1992. There were 14,062 live births and 13,988 children were alive at age 1 year. When the oldest children were approximately 7 years of age, an attempt was made to bolster the initial sample with eligible cases who had failed to join the study originally resulting in 15,454 pregnant women who had a total of 15,589 fetuses, of which 14,901 children alive at 1 year of age (Figure 1). Regular clinic visits of the children commenced at 7 years of age and are still ongoing. Please note that the study website contains details of all the data that is available through a fully searchable data dictionary and variable search tool http://www.bristol.ac.uk/alspac/researchers/our-data/ and in published studies.[1-3] In the cross-sectional analyses we included 1799 participants who had complete measurement data for total fat mass, trunk fat mass, lean mass, height, weight, blood pressure, cfPWV, and cIMT during age 24-year follow-up clinic visit. In the prospective analyses, we included 3863 participants who had complete clinic measurements for cfPWV and cIMT at age 17-year follow-up clinic visit. The demographic characteristics of excluded participants were similar to those included in this study (Supplementary Table 1). Ethical approval for the study was obtained from the ALSPAC Ethics and Law Committee and the Local Research Ethics Committees. Informed consent for the use of data collected via questionnaires and clinics was obtained from participants following the recommendations of the ALSPAC Ethics and Law Committee at the time. At age ≥16 years, participants personally provided their informed written consent. Consent for biological samples has been collected in accordance with the Human Tissue Act (2004). Study data at 24 years were collected and managed using REDCap electronic data capture tools.[4,5]

### Anthropometry and body composition

Anthropometry (height and weight) at ages 9 and 17 years was assessed using standard protocols.[6] At 24 years of age, standing height to the nearest millimeters was measured using a Harpenden wall-mounted stadiometer (Holtain Ltd, Crosswell, Crymych, UK). Weight to the nearest 0.1kg at age 24 years was measured using Tanita TBF-401 (Model A, Tanita Corp., Tokyo, Japan) electronic body composition scales. At ages 9 and 17 years, body composition was assessed using dual-energy Xray absorptiometry (DEXA) scanner as earlier described.[6,7] At 24 years, body composition was measured using DEXA scanner (Lunar Prodigy software version 15, GE Medical Systems, Madison, Wisconsin). Repeated DEXA measurements for 122 children were performed on the same day, and the repeatability coefficient (twice the standard deviation of the difference between measurement occasions) for body fat mass was 0.5 kg. [6,7] We calculated body mass index (BMI) by dividing weight by squared height, total and trunk fat mass indices (FMI) as the ratio of total and trunk fat mass to squared height, and lean mass index (LMI) as the ratio of lean mass to squared height.[8] BMI was also categorized as normal weight if <24.99 kg/$m^2$ and overweight and obese if >24.99 kg/$m^2$.

### Vascular phenotype

At age 17 years clinic visit, cfPWV arterial measure was recorded three times. For the cfPWV, a cuff was placed over the right carotid artery in the participant's neck, while another was located

over the femoral artery in their upper right thigh. The distance between the participant's suprasternal notch and the top of the thigh cuff was measured, as was the distance between their suprasternal notch and the bottom of the neck cuff on the right side. cfPWV and transit time to the nearest 0.01 ms were automatically computed from measurements of pulse transit time and distance travelled by the pulse between two recording sites using Vicorder (Skidmore Medical, Bristol, UK) portable physiologic vascular testing equipment. All measurements were taken independently by one of two trained vascular technicians (inter-observer mean difference 0·2 m/sec, SD 0·1).[6,7] At 24 years, cfPWV was measured, five minutes after resting in a semi-prone position, using a Vicorder instrument (Skidmore Medical, Bristol, UK) with two blood pressure measurement channels and two Velcro pressure sensor cuffs applied over each of the carotid and femoral arteries. The cfPWV measurement was repeated until three readings that were within 0.5 m/sec of each other had been recorded. Age- and sex-specific 90th percentile of cfPWV indicates high risk of subclinical arteriosclerosis.

cIMT was assessed by ultrasound using a linear 12-MHz transducer (Vivid7, GE Medical, Chicago, Illinois) as earlier reported.[6,7] The average of cIMT at 17 years serially measured at 3 different cardiac cycles was computed. Interobserver variability for cIMT was assessed in a separate sample of 25 young adults (coefficient of variation: 4.4 ± 2.2%).[6,7] The right and left common carotid arteries at age 24 years were imaged using an ultrasound machine (CardioHealth Panasonic and a 13.5 MHz linear array broadband transducer (probe; centre frequency 9.0 MHz).[7,9] Participants were placed in a supine position with the head rotated by 45 degrees from the midpoint. An automated guide line was placed at the bulb (a longitudinal scan that included the common carotid artery and the carotid bifurcation) with the region-of-interest box and IMT trace lines automatically positioned 1 cm away from the guide line. The scanner automatically saved an image when the region-of-interest box turned green, indicating good image quality. An automated cIMT measurement, recorded from the posterior wall of the artery, was saved after three consecutive cardiac cycles. When interrogating the common carotid, the CardioHealth system calculated and displayed the cIMT that is updated at each detected R-wave of the cardiac cycle. Once the measurement achieved a predefined quality threshold, scanning automatically stopped and a report was generated. Raw data were checked for outliers and cIMT value >1.0 mm was reviewed by a trained research scientist to assess validity. Abnormal values due to measurement error were removed. Participants had between 1 to 3 cIMT measures for each of the right and left carotid arteries. For our analysis, we computed the mean of the average measurement of the right and left common carotid arteries as cIMT. Age- and sex-specific 90th percentile of cIMT indicates a high risk of subclinical atherosclerosis.

**Cardiometabolic and lifestyle factors**

Pulse rate and BP were measured at ages 9 and 17 years as previously detailed.[6,7] BP readings at the 24-year clinic visit were taken using an Omron M6 upper arm BP/pulse monitor. Participants were asked to sit and rest for two minutes prior to taking the first seated BP reading. Participants were categorized as normotensive if systolic BP is <120 mm Hg and elevated BP or hypertension when systolic BP is >120 mmHg. Only 6 participants received anti-hypertensive medication at age 17 years. Using standard protocols, blood samples at ages 9, 17, and 24 years were collected, spun, and frozen at –80 °C and a detailed assessment of fasting glucose, insulin, high sensitivity C-reactive protein (hsCRP), low-density lipoprotein cholesterol (LDL-C), high-density lipoprotein cholesterol, and triglycerides, has been reported (coefficient of variation was <5%).[7,10]

Questionnaire to assess smoking behavior were administered at the 17-year[6] and 24-year clinic visits. The participants were asked whether they smoked in the last 30 days, smoked a whole cigarette, smoked every day, their frequency of use, etc. At the 17-year clinic visit, participants were briefly asked about their personal and family (mother, father, and siblings) medical history such as, a history of hypertension, diabetes, high cholesterol, and vascular disease. Physical activity at age 24 years was assessed using ActiGraph GT3X+ accelerometer device worn for four

consecutive days, ideally starting the day after the clinic visit. Moderate to vigorous physical activity reported in minutes per day was based on a previously established cutoff of >2020 count per minute.[11] Valid days were considered as wear time of at least 500 minutes, after excluding intervals of >60 minutes of zero counts.

**Missing data and multiple imputations**

Eligible sample size varied by predictor and outcome measure, as presented in Supplementary Tables S2 and S3. Exclusions via listwise deletion of missing values ranged from 15.6 to 63.9 percent for covariates. We restricted study participants to those who had complete outcome variables at age 17 years follow-up (n=3863) and complete predictors and outcome variables at 24 years of age follow-up (n=1799). We conducted a Little's missing completely at random (MCAR) test to ascertain data missingness.[12] Little's MCAR test: Chi-Square = 683.366, Degree of freedom = 409, P-value <0.0001, made us conclude that the variables are not missing completely at random. Regression-modeled multiple imputations were conducted using SPSS version 27 (IBM Corp, Armonk, NY, USA). Imputed variables are described in Supplementary Tables 4 and 5. The observed minimum and maximum values were constraints for the imputation process and 20 cycles of imputation with 10 iterations resulted in 20 imputed data sets. The multiple imputation module in SPSS pooled the results from these imputed data. In line with previous evidence (3), the percentage of missing values would be sufficiently addressed with 20 imputations: the variable with the highest missing value (63.9%, moderate to vigorous physical activity) had an estimate that was 98% efficient after 20 imputations (computed using Rubin's formula).[12] The distributions of imputed covariates had the same pattern as in the observed data as evidenced in the histogram normality plot. Variable distributions after imputation (mean, SD, and percentages) were similar to the observed data (Supplementary Table 6). Imputed results of cross-sectional and longitudinal analyses are presented in the main article while for sensitivity analysis the non-imputed results are presented in Supplementary Tables 7 – 9. Any inconsistencies between the complete case analysis and imputed results is due to a significantly low number of study participants (circa 8%) who had complete predictors, outcomes, and covariates of interest. Where multiple imputations have been conducted, presenting imputed results is preferred over presenting non-imputed results (pairwise or listwise deletion).[13]

**Statistical Analysis**

Participant's descriptive characteristics were summarized as means and standard deviation, medians and interquartile ranges, or frequencies and percentages, presented in the main article and in Supplementary Tables 10-13. We explored sex differences using Independent t-tests, Mann Whitney-U tests, or Chi-square tests for normally distributed, skewed or dichotomous variables, respectively. We assessed the normality of variables by histogram curve, quantile-quantile plot, and Kolmogorov-Smirnov tests. We conducted a logarithmic transformation of skewed variables and confirmed normality prior to further analysis.

We examined the separate cross-sectional associations of total fat mass, total FMI, trunk fat mass, trunk FMI, lean mass, LMI, BMI, or BP (predictors) with cfPWV and cIMT (outcomes) at age 24 years using multiple linear regression analyses. We presented univariate analysis, adjusting for sex, and multivariate analysis adjusted for age, sex, LDL-C, hsCRP, fasting blood glucose, systolic BP, fat mass and/or lean mass depending on the predictor, moderate to vigorous physical activity, smoking status and family history of hypertension/diabetes/high cholesterol/vascular disease. For sensitivity analysis, we investigated the cross-sectional associations of categories of the above-named predictors, divided in quartiles according to age- and population-specific distribution of low [≤24.9%], moderate-low [25 – 49.9%], moderate-high [50 – 74.9%], and very high [≥75%], with the outcomes. We investigated across group differences using generalized linear multivariable-adjusted analyses with Sidak correction for multiple comparisons while one-way analysis of variance was used to examine the linear trend.

To examine the separate cumulative effects of the predictors measured from ages 9 – 24 years on the 7-year change in cfPWV and cIMT from adolescence to adulthood, we used linear mixed-effects models with restricted maximum likelihood estimation. Each tertile category were cumulatively combined with a similar category for each age during data restructure before conducting the linear mixed-effect model analyses. The restructured data reflects the lowest tertile at each of age 9, 17. and 24 years as the reference, in relation to moderate and high tertiles categories for each age 9, 17, and 24 years. We indexed fat mass and lean mass to squared height to account for inter-individual growth variation across the follow-up period (14.65 ± 0.82 years). The estimates quantify the effect of cumulative exposures to the predictors on the outcome variables. We decided a priori to select the model with the least Bayesian information criterion (BIC). The least BIC resulted in a model with sex as a factor and a random intercept modeled on the subject level. We selected a variance component covariance type and determined the effect of the predictors on the outcome variables. We classified the predictors based on age- and population-specific tertiles (3 equal distribution); low, moderate, and high categories and computed two models per outcome. Model 1 was adjusted for sex, age at baseline, time i.e difference in years (continuous variable) between baseline measure at age 9 years and that at age 24 years follow-up, and cardiometabolic factors such as systolic BP at ages 9, 17, and 24 years, LDL-C at ages 9, 17, and 24 years, hsCRP at ages 9, 17, and 24 years, fasting blood glucose at ages 17 and 24 years, and fat mass and/or lean mass at ages 9, 17, and 24 years depending on the predictor. Model 2 was a further adjustment of model 1 for lifestyle factors viz, moderate to vigorous physical activity at age 24 years, smoking status at ages 17 and 24 years, and family history of hypertension/diabetes/high cholesterol/vascular disease. We did not adjust the models for heart rate due to high collinearity (r = 0.87) with systolic blood pressure. Also, height was not adjusted for because we observed near-perfect collinearity with lean mass (r = 0.95). For sensitivity analyses, we also presented results based on BMI categories of normal weight status (<24.99 kg/m$^2$) and overweight and obesity status (>24.99 kg/m$^2$) in Supplementary Table 14.

Furthermore, we examined the separate effect of cumulative exposures to predictors on cfPWV and cIMT at 24 years of age, with a similar adjustment strategy, using the linear mixed/effect model as detailed above. However, the best model fit for this analysis did not include the random intercept. We also investigated the role of atherosclerosis (≥90th percentile cIMT) and vascular aging from elastin degradation (≥90th percentile cfPWV) on the cumulative effect of total fat mass, lean mass, and systolic blood pressure on carotid-femoral pulse wave velocity and carotid intima-media thickness progression with a similar adjustment strategy, using the linear mixed-effect model as detailed above. All covariates were selected based on previous studies.[6-8,14,15] We excluded pubertal status/somatic maturation from the model because, at the study baseline (9 years of age), only 0.5% had attained maturity while all participants had reached adult-like maturity status by 17 years of age follow-up.

We performed collinearity diagnoses and accepted results with a variance inflation factor <5. There was no statistically significant sex by predictor interaction with cIMT and a few sex-by-predictor interactions with cfPWV, hence we presented combined results and adjusted for sex. We also reported all significant sex-based results that differed from the combined analyses. We considered differences and associations with a 2-sided p-value <0.05 as statistically significant and made conclusions based on effect estimates and their confidence intervals (CI). Analyses involving 40% of a sample of 10,000 ALSPAC children at 0.8 statistical power, 0.05 alpha, and 2-sided p-value would show a minimum detectable effect size of 0.049 standard deviation if they had relevant exposure for a normally distributed quantitative variable.[16] All statistical analyses were performed using SPSS statistics software, Version 27.0 (IBM Corp, Armonk, NY, USA).

## References


1. Boyd A, Golding J, Macleod J, Lawlor DA, Fraser A, Henderson J, Molloy L, Ness A, Ring S, Davey Smith G. Cohort Profile: the 'children of the 90s'--the index offspring of the Avon Longitudinal Study of Parents and Children. *Int J Epidemiol*. 2013;42:111-127. doi:10.1093/ije/dys064

2. Fraser A, Macdonald-Wallis C, Tilling K, Boyd A, Golding J, Davey Smith G, Henderson J, Macleod J, Molloy L, Ness A, Ring S, Nelson SM, Lawlor DA. Cohort Profile: the Avon Longitudinal Study of Parents and Children: ALSPAC mothers cohort. *Int J Epidemiol*. 2013;42:97-110. doi:10.1093/ije/dys066

3. Northstone K, Lewcock M, Groom A, Boyd A, Macleod J, Timpson NJ, Wells N. The Avon Longitudinal Study of Parents and Children (ALSPAC): an updated on the enrolled sample of index children in 2019. Wellcome Open research 2019;4:51 (https://doi.org/10.12688/wellcomeopenres.15132.1)

4. Harris PA, Taylor R, Thielke R, Payne J, Gonzalez N, Conde JG. Research electronic data capture (REDCap)--a metadata-driven methodology and workflow process for providing translational research informatics support. *J Biomed Inform*. 2009;42:377-381. doi:10.1016/j.jbi.2008.08.010

5. Harris PA, Taylor R, Minor BL, Elliott V, Fernandez M, O'Neal L, McLeod L, Delacqua G, Delacqua F, Kirby J, Duda SN; REDCap Consortium. The REDCap consortium: The REDCap consortium: Building an international community of software platform partners. *J Biomed Inform*. 2019;95:103208. doi:10.1016/j.jbi.2019.103208

6. Agbaje AO, Barker AR, Tuomainen TP. Effects of arterial stiffness and carotid intima-media thickness progression on the risk of overweight/obesity and elevated blood pressure/hypertension: a cross-lagged cohort study. Hypertension. 2022;79(1):159-169.

7. Agbaje AO, Barker AR, Mitchell GF, Tuomainen TP. Effect of Arterial Stiffness and Carotid Intima-Media Thickness Progression on the Risk of Dysglycemia, Insulin Resistance, and Dyslipidemia: a Temporal Causal Longitudinal Study. Hypertension. 2022;79(3):667-678.

8. Sletner L, Mahon P, Crozier SR, Inskip HM, Godfrey KM, Chiesa S, Bhowruth DJ, Charakida M, Deanfield J, Cooper C, Hanson M. Childhood Fat and Lean Mass: Differing Relations to Vascular Structure and Function at Age 8 to 9 Years. *Arterioscler Thromb Vasc Biol*. 2018;38:2528-2537. doi:10.1161/ATVBAHA.118.311455

9. Vanoli D, Wiklund U, Lindqvist P, Henein M, Näslund U. Successful novice's training in obtaining accurate assessment of carotid IMT using an automated ultrasound system. *Eur Heart J Cardiovasc Imaging*. 2014;15:637-642. doi:10.1093/ehjci/jet254

10. Falaschetti E, Hingorani AD, Jones A, Charakida M, Finer N, Whincup P, Lawlor DA, Davey Smith G, Sattar N, Deanfield JE. Adiposity and cardiovascular risk factors in a large contemporary population of pre-pubertal children. *Eur Heart J*. 2010;31:3063-3072. doi:10.1093/eurheartj/ehq355

11. Troiano RP, Berrigan D, Dodd KW, Mâsse LC, Tilert T, McDowell M. Physical activity in the United States measured by accelerometer. *Med Sci Sports Exerc*. 2008;40:181-188. doi:10.1249/mss.0b013e31815a51b3

12. Rubin DB. An Overview of Multiple Imputation. *Proc Surv Res methods Sect Am Stat Assoc*. 1988.

13. Mackinnon A. The use and reporting of multiple imputation in medical research - a review. *J Intern Med*. 2010;268:586-593. doi:10.1111/j.1365-2796.2010.02274.x

14. Wade KH, Chiesa ST, Hughes AD, et al. Assessing the causal role of body mass index on cardiovascular health in young adults: Mendelian randomization and recall-by-genotype analyses. *Circulation*. 2018;138:2187-2201. doi:10.1161/CIRCULATIONAHA.117.033278

15. Zhang Y, Vittinghoff E, Pletcher MJ, et al. Associations of Blood Pressure and Cholesterol Levels During Young Adulthood With Later Cardiovascular Events. *J Am Coll Cardiol*. 2019;74:330-341. doi:10.1016/j.jacc.2019.03.529

16. Golding J, Pembrey M, Jones R; ALSPAC Study Team. ALSPAC--the Avon Longitudinal Study of Parents and Children. I. Study methodology. *Paediatr Perinat Epidemiol*. 2001;15:74-87. doi:10.1046/j.1365-3016.2001.00325.x

## Supplementary Results

**Supplementary Table 1** Characteristics of participants excluded from the study

| Variables | Included participants | | Excluded participants | | | |
|---|---|---|---|---|---|---|
| | **Mean (SD)** | **n** | **Mean/SD** | **n** | ***P* for difference** | **Cohen's D** |
| Age (years) | 17.72 (0.32) | 3863 | 18.12 (0.61) | 1349 | **<0.0001** | 0.82 |
| ***Anthropometry and body composition*** | | | | | | |
| Body height (m) | 1.71 (0.09) | 3806 | 1.71 (0.10) | 1259 | **0.007** | 0 |
| *Weight (kg) | 64.40 (142.6) | 3811 | 66.30 (119.3) | 1253 | **<0.0001** | 0.25 |
| *Body mass index (kg/m$^2$) | 21.78 (52.1) | 3806 | 22.69 (46.9) | 1253 | **<0.0001** | 0.32 |
| Lean mass (kg) | 45.53 (9.88) | 3757 | 45.72 (10.47) | 1090 | 0.584 | NA |
| *Total fat mass (kg) | 16.01 (80.8) | 3757 | 17.70 (71.3) | 1090 | **<0.0001** | 0.29 |
| *Trunk fat mass (kg) | 7.88 (45.5) | 3757 | 8.87 (40.6) | 1090 | **<0.0001** | 0.29 |
| ***Metabolic profile*** | | | | | | |
| Total cholesterol (mmol/L) | 3.75 (0.68) | 2586 | 3.77 (0.70) | 699 | 0.422 | NA |
| HDL (mmol/L) | 1.27 (0.30) | 2586 | 1.27 (0.30) | 699 | 0.911 | NA |
| LDL (mmol/L) | 2.10 (0.61) | 2586 | 2.11 (0.61) | 699 | 0.740 | NA |
| *Triglyceride (mmol/L) | 0.75 (3.47) | 2586 | 0.77 (3.89) | 699 | 0.065 | 0.10 |
| *C-reactive protein (mg/L) | 0.54 (1.01) | 2586 | 0.61 (0.40) | 699 | **0.039** | 0.06 |
| *Insulin (mU/L) | 6.67 (89.1) | 2543 | 6.94 (193.6) | 688 | **0.001** | 0.19 |
| Glucose (mmol/L) | 5.04 (0.62) | 2586 | 5.04 (0.46) | 699 | 0.770 | NA |
| ***Vascular measure*** | | | | | | |
| Pulse rate (beats/mins) | 65 (10) | 3854 | 66 (10) | 809 | 0.197 | NA |
| Systolic BP (mm Hg) | 114 (10) | 3854 | 114 (11) | 809 | 0.694 | NA |
| Diastolic BP (mm Hg) | 64 (6) | 3854 | 66 (7) | 809 | **<0.0001** | 0.31 |
| *Carotid IMT (mm) | 0.47 (0.35) | 3861 | 0.48 (0.27) | 815 | 0.807 | NA |
| *Carotid-femoral PWV (m/s) | 5.70 (7.68) | 3857 | 5.62 (3.54) | 26 | 0.389 | NA |
| ***Lifestyle factors*** | | | | | | |
| Smoking status (n, %) | 911 (27.2) | 3344 | 268 (31.4) | 853 | **0.017** | NA |
| Family history of HDCV (n, %) | 1162 (30.1) | 3857 | 289 (31.2) | 927 | 0.533 | NA |

The values are means (standard deviations) and *median (range/interquartile range) except for maturation status and social economic status in percentage. Differences between participants were tested using Student's t-test for normally distributed continuous variables, Mann–Whitney U test for skewed continuous variables, and Chi-square test for dichotomous variable. A 2-sided P-value <0.05 is considered statistically significant and is bolded. Cohen's D effect size was calculated for statistically significant differences: 0.2 = low, 0.5 = moderate, 0.8 = large effect.

HDCV, hypertension/diabetes/high cholesterol/vascular disease; IMT, intima media thickness; NA, not applicable; PWV, pulse wave velocity; Smoking status, participants had smoked cigarette in the past 30 days.

**Supplementary Table 2** Missing data at 24 years of age

| Variable | n (valid sample size) | Eligible sample size | % Missing |
|---|---|---|---|
| Age | 1799 | 1799 | 0 |
| Sex | 1799 | 1799 | 0 |
| ***Anthropometry and body composition*** | | | |
| Height (m) | 1799 | 1799 | 0 |
| Weight (kg) | 1799 | 1799 | 0 |
| Body mass index | 1799 | 1799 | 0 |
| Lean mass (kg) | 1799 | 1799 | 0 |
| Lean mass indexed for squared height (kg/m$^2$) | 1799 | 1799 | 0 |
| Total fat mass (kg) | 1799 | 1799 | 0 |
| Total fat mass indexed for squared height (kg/m$^2$) | 1799 | 1799 | 0 |
| Trunk fat mass (kg) | 1799 | 1799 | 0 |
| Trunk fat mass indexed for squared height (kg/m$^2$) | 1799 | 1799 | 0 |
| ***Metabolic profile*** | | | |
| Low-density lipoprotein (mmol/L) | 1490 | 1799 | 17.2 |
| Glucose (mmol/L) | 1491 | 1799 | 17.1 |
| C-reactive protein (mg/L) | 1352 | 1799 | 24.8 |
| ***Vascular measure*** | | | |
| Systolic blood pressure (mm Hg) | 1799 | 1799 | 0 |
| Diastolic blood pressure (mm Hg) | 1799 | 1799 | 0 |
| Carotid-femoral pulse wave velocity (m/s) | 1799 | 1799 | 0 |
| Carotid intima-media thickness (mm) | 1799 | 1799 | 0 |
| ***Lifestyle factors*** | | | |
| Moderate to vigorous physical activity (mins/day) | 649 | 1799 | 63.9 |
| Smoking status | 1779 | 1799 | 1.1 |
| Family history of H-C-D-V | 1519 | 1799 | 15.6 |

H-D-C-V, hypertension/diabetes/high cholesterol/vascular disease

**Supplementary Table 3** Missing data at 17 years of age

| Variable | n (valid sample size) | Eligible sample size | % Missing |
|---|---|---|---|
| Age | 3863 | 3863 | 0 |
| Sex | 3863 | 3863 | 0 |
| ***Anthropometry and body composition*** | | | |
| Height (m) | 3806 | 3863 | 1.5 |
| Weight (kg) | 3811 | 3863 | 1.3 |
| Body mass index | 3806 | 3863 | 1.5 |
| Lean mass (kg) | 3756 | 3863 | 2.8 |
| Lean mass indexed for squared height ($kg/m^2$) | 3737 | 3863 | 3.3 |
| Total fat mass (kg) | 3756 | 3863 | 2.8 |
| Total fat mass indexed for squared height ($kg/m^2$) | 3737 | 3863 | 3.3 |
| Trunk fat mass (kg) | 3756 | 3863 | 2.8 |
| Trunk fat mass indexed for squared height ($kg/m^2$) | 3737 | 3863 | 3.3 |
| ***Metabolic profile*** | | | |
| Low-density lipoprotein (mmol/L) | 2587 | 3863 | 33.0 |
| Glucose (mmol/L) | 2587 | 3863 | 33.0 |
| C-reactive protein (mg/L) | 2587 | 3863 | 33.0 |
| ***Vascular measure*** | | | |
| Systolic blood pressure (mm Hg) | 3856 | 3863 | 0.2 |
| Diastolic blood pressure (mm Hg) | 3856 | 3863 | 0.2 |
| Carotid-femoral pulse wave velocity (m/s) | 3863 | 3863 | 0 |
| Carotid intima-media thickness (mm) | 3863 | 3863 | 0 |
| ***Lifestyle factors*** | | | |
| Smoking status | 3344 | 3863 | 27.2 |
| Family history of H-C-D-V | 3859 | 3863 | 0.1 |

H-D-C-V, hypertension/diabetes/high cholesterol/vascular disease

**Supplementary Table 4** Variables used in multivariable multiple imputation model at 24 years

| Variable | Missing values imputed in this variable | Imputed values | | | |
|---|---|---|---|---|---|
| | | Minimum | Maximum | Rounding | (n) imputed |
| Age (years) | NA, no missing | | | Integer | |
| Sex | NA, no missing | | | | |
| ***Anthropometry and body composition*** | | | | | |
| Height (m) | NA, no missing | | | | |
| Weight (kg) | NA, no missing | | | | |
| Body mass index (kg/m$^2$) | NA, no missing | | | | |
| Lean mass (kg) | NA, no missing | | | | |
| Lean mass indexed for squared height (kg/m$^2$) | NA, no missing | | | | |
| Total fat mass (kg) | NA, no missing | | | | |
| Total fat mass indexed for squared height (kg/m$^2$) | NA, no missing | | | | |
| Trunk fat mass (kg) | NA, no missing | | | | |
| Trunk fat mass indexed for squared height (kg/m$^2$) | NA, no missing | | | | |
| ***Metabolic profile*** | | | | | |
| Low-density lipoprotein (mmol/L) | Yes | 0.15 | 5.73 | | 309 |
| Glucose (mmol/L) | Yes | 3.59 | 22.15 | | 308 |
| C-reactive protein (µg/ml) | Yes | 0.10 | 164.42 | | 447 |
| ***Vascular measure*** | | | | | |
| Systolic blood pressure (mm Hg) | NA, no missing | | | | |
| Diastolic blood pressure (mm Hg) | NA, no missing | | | | |
| Carotid-femoral pulse wave velocity (m/s) | NA, no missing | | | | |
| Carotid intima media thickness (mm) | NA, no missing | | | | |
| ***Lifestyle factors*** | | | | | |
| Moderate to vigorous physical activity | Yes | 3.17 | 181.06 | | 1150 |
| Smoking status | Yes | 0 | 1 | | 20 |
| Family history of H-D-C-V | Yes | 1 | 2 | | 280 |

H-D-C-V, hypertension/diabetes/high cholesterol/vascular disease; NA, not applicable

**Supplementary Table 5** Variables used in multivariable multiple imputation model at 17 years

| Variable | Missing values imputed in this variable | Imputed values | | | |
|---|---|---|---|---|---|
| | | Minimum | Maximum | Rounding | (n) imputed |
| Age (years) | NA, no missing | 16.25 | 19.92 | Integer | 0 |
| Sex | NA, no missing | | | | 0 |
| ***Anthropometry and body composition*** | | | | | |
| Height (m) | Yes | 1.47 | 2.08 | | 57 |
| Weight (kg) | Yes | 21.40 | 144.0 | | 52 |
| Body mass index (kg/m$^2$) | Yes | 9.36 | 41.49 | | 57 |
| Lean mass (kg) | Yes | 23.72 | 74.81 | | 107 |
| Lean mass indexed for squared height (kg/m$^2$) | Yes | 7.27 | 21.87 | | 126 |
| Total fat mass (kg) | Yes | 1.68 | 65.56 | | 107 |
| Total fat mass indexed for squared height (kg/m$^2$) | Yes | 0.51 | 20.77 | | 126 |
| Trunk fat mass (kg) | Yes | 0.96 | 38.01 | | 107 |
| Trunk fat mass indexed for squared height (kg/m$^2$) | Yes | 0.30 | 11.62 | | 126 |
| ***Metabolic profile*** | | | | | |
| Low-density lipoprotein (mmol/L) | Yes | 0.23 | 5.10 | | 1276 |
| Glucose (mmol/L) | Yes | 3.44 | 8.66 | | 1276 |
| C-reactive protein (µg/ml) | Yes | 0.20 | 86.47 | | 1276 |
| ***Vascular measure*** | | | | | |
| Systolic blood pressure (mmnHg) | Yes | 82.5 | 152.0 | | 7 |
| Diastolic blood pressure (mm Hg) | Yes | 42.3 | 104.0 | | 7 |
| Carotid-femoral pulse wave velocity (m/s) | NA, no missing | 4.27 | 11.46 | | 0 |
| Carotid intima media thickness (mm) | NA, no missing | 0.35 | 0.63 | | 0 |
| ***Lifestyle factors*** | | | | | |
| Smoking status | Yes | 1 | 2 | | 518 |
| Family history of H-D-CV | Yes | 1 | 2 | | 4 |

H-D-CV, hypertension/diabetes/high cholesterol/vascular disease; NA, not applicable

**Supplementary Table 6** Characteristics of cohort participants at age 24 years (imputed data)

| **Variable** | **Observed data set Mean/SD** | **Imputed data set Mean/SD** |
|---|---|---|
| Age | 24.32 (0.63) | NA |
| Sex, male (n,%) | 684 (38) | NA |
| ***Anthropometry and body composition*** | | |
| Height (m) | 1.72 (0.09) | NA |
| Weight (kg) | | NA |
| [a]Body mass index (kg/m$^2$) | 24.62 (4.68) | NA |
| [a]Lean mass (kg) | 47.21 (9.79) | NA |
| [a]Lean mass indexed for squared height (kg/m$^2$) | 15.91 (2.17) | NA |
| [a]Total fat mass (kg) | 22.91 (10.16) | NA |
| [a]Total fat mass indexed for squared height (kg/m$^2$) | 7.88 (3.61) | NA |
| [a]Trunk fat mass (kg) | 10.99 (5.86) | NA |
| [a]Trunk fat mass indexed for squared height (kg/m$^2$) | 3.77 (2.04) | NA |
| ***Metabolic profile*** | | |
| Low-density lipoprotein (mmol/L) | 2.38 (0.74) | 2.40 (0.74) |
| Glucose (mmol/L) | 5.33 (0.70) | 5.32 (0.70) |
| [a]C-reactive (mg/L) | 2.19 (6.00) | 2.79 (5.83) |
| ***Vascular measure*** | | |
| Systolic blood pressure (mm Hg) | 115 (11) | NA |
| Diastolic blood pressure (mm Hg) | 67 (8) | NA |
| [a]Carotid-femoral pulse wave velocity (m/s) | 6.27 (1.08) | NA |
| [a]Carotid intima media thickness (mm) | 0.46 (0.05) | NA |
| ***Lifestyle factors*** | | |
| Moderate to vigorous physical activity | 49.60 (29.9) | 52.0 (29.1) |
| Smoked cigarette in the last 30 days, Yes, (n,%) | 455 (25.6) | 20 (1.1) |
| Family history of H-C-D-V, Yes, (n,%) | 473 (31.1) | 559 (31.1) |

H-D-C-V, hypertension/diabetes/high cholesterol/vascular disease; NA, Not applicable; [a]skewed variables presented as median and inter quartile range

**Supplementary Table 7** Cross-sectional association of fat mass, lean mass and systolic blood pressure with carotid-femoral pulse wave velocity and carotid intima media thickness at age 24 years. **(Non-imputed data, pairwise deletion)**

| **N= 1799** for predictors and outcomes | **Carotid-femoral pulse wave velocity (m/s)** | | | | **Carotid intima-media thickness (mm)** | | | |
|---|---|---|---|---|---|---|---|---|
| | Univariate analysis | | Multivariate analysis | | Univariate analysis | | Multivariate analysis | |
| | *β* (95% CI) | p-value | *β* (95% CI) | p-value | *β* (95% CI) | p-value | *β* (95% CI) | p-value |
| Total fat mass (kg) | 0.029 (0.011 – 0.046) | **0.001** | -0.006 (-0.048 – 0.036) | 0.775 | 0.004 (-0.008 – 0.015) | 0.547 | -0.030 (-0.057 – -0.002) | **0.035** |
| Trunk fat mass (kg) | 0.020 (0.005 – 0.034) | **0.007** | -0.010 (-0.043 – 0.024) | 0.578 | 0.0001 (-0.009 – 0.010) | 0.978 | -0.026 (-0.048 – -0.004) | **0.022** |
| Lean mass (kg) | 0.160 (0.107 – 0.213) | **<0.0001** | 0.116 (-0.020 – 0.235) | 0.054 | 0.132 (0.097 – 0.166) | **<0.0001** | 0.143 (0.065 – 0.222) | **<0.0001** |
| Total fat mass index (kg/m$^2$) | 0.018 (0.0001 – 0.035) | **0.045** | -0.015 (-0.055 – 0.025) | 0.465 | 0.0001 (-0.012 – 0.011) | 0.970 | -0.027 (-0.054 – <0.0001) | **0.047** |
| Trunk fat mass index (kg/m$^2$) | 0.012 (-0.002 – 0.027) | 0.088 | -0.015 (-0.048 – 0.017) | 0.363 | -0.002 (-0.012 – 0.007) | 0.620 | -0.024 (-0.046 – -0.002) | **0.029** |
| Lean mass index (kg/m$^2$) | 0.085 (0.021 – 0.149) | **0.009** | -0.007 (-0.149 – 0.135) | 0.924 | 0.138 (0.096 – 0.180) | **<0.0001** | 0.146 (0.052 – 0.240) | **0.002** |
| Body mass index (kg/m$^2$) | 0.058 (0.018 – 0.097) | **0.004** | -0.071 (-0.178 – 0.036) | 0.195 | 0.040 (0.013 – 0.066) | **0.003** | -0.053 (-0.124 – 0.018) | 0.144 |
| Systolic blood pressure (mm Hg) | 0.001 (0.0001 – 0.001) | **<0.0001** | 0.010 (<0.0001 – 0.001) | 0.052 | 0.001 (0.0001 – 0.001) | **<0.0001** | 0.001 (<0.0001 – 0.001) | **0.001** |
| Diastolic blood pressure (mm Hg) | 0.001 (0.001 – 0.002) | **<0.0001** | 0.001 (0.0001 – 0.002) | **0.003** | 0.0001 (-0.0001 – 0.001) | 0.212 | 0.0001 (<0.0001 – 0.001) | 0.527 |

Univariate analysis was adjusted for sex. Multivariate analysis was adjusted for age, sex, low density lipoprotein, C-reactive protein, fasting blood glucose, systolic blood pressure and fat mass and/or lean mass depending on the predictor, moderate to vigorous physical activity at 24 years, smoking status and family history of hypertension/diabetes/high cholesterol/vascular disease (H-D-C-V). Sample size for predictors and outcomes was 1799, but some covariates had varying sample sizes ranging from 649, moderate to vigorous physical activity to 1549, family history of H-D-C-V. Predictors and outcomes were skewed variables but were logarithmically transformed before linear regression analyses. *β* is unstandardized regression coefficient, CI, confidence interval. Associations with p-value <0.05 are considered statistically significant.

**Supplementary Table 8** Cumulative effect of exposure to fat mass, lean mass, and systolic blood pressure from 9 to 24 years on carotid-femoral pulse wave velocity and carotid intima media thickness at age 24 years (Non-imputed dataset, listwise deletion)

| **N=182** | **Carotid-femoral pulse wave velocity** | | | | **Carotid intima-media thickness** | | | |
|---|---|---|---|---|---|---|---|---|
| | **Model 1** | | **Model 2** | | **Model 1** | | **Model 2** | |
| | Effect estimate (95% CI) | p-value | Effect estimate (95% CI) | p-value | Effect estimate (95% CI) | p-value | Effect estimate (95% CI) | p-value |
| **Total fat mass (kg)** | | | | | | | | |
| *Low category* | Reference | – | Reference | – | Reference | – | Reference | – |
| *Moderate category* | 0.004 (-0.017 – 0.025) | 0.724 | 0.002 (-0.019 – 0.024) | 0.833 | 0.011 (-0.001 – 0.023) | 0.061 | 0.011 (-0.002 – 0.015) | 0.129 |
| *High category* | -0.002 (-0.024 – 0.021) | 0.892 | -0.005 (-0.028 – 0.018) | 0.686 | 0.007 (-0.006 – 0.020) | 0.279 | 0.005 (-0.008 – 0.018) | 0.423 |
| **Trunk fat mass (kg)** | | | | | | | | |
| *Low category* | Reference | – | Reference | – | Reference | – | Reference | – |
| *Moderate category* | -0.002 (-0.023 – 0.019) | 0.877 | -0.004 (-0.025 – 0.017) | 0.719 | 0.007 (-0.005 – 0.018) | 0.268 | 0.006 (-0.006 – 0.018) | 0.344 |
| *High category* | 0.001 (-0.021 – 0.023) | 0.944 | -0.003 (-0.025 – 0.020) | 0.828 | 0.0001 (-0.012 – 0.012) | 0.987 | -0.002 (-0.014 – 0.011) | 0.802 |
| **Lean mass (kg)** | | | | | | | | |
| *Low category* | Reference | – | Reference | – | Reference | – | Reference | – |
| *Moderate category* | -0.005 (-0.025 – 0.016) | 0.640 | 0.003 (-0.011 – 0.032) | 0.684 | 0.013 (0.002 – 0.024) | **0.025** | 0.012 (0.004 – 0.020) | **0.004** |
| *High category* | 0.009 (-0.018 – 0.037) | 0.504 | 0.015 (-0.002 – 0.032) | 0.076 | 0.026 (0.011 – 0.040) | **0.001** | 0.016 (0.005 – 0.026) | **0.003** |
| **Total fat mass index (kg/m$^2$)** | | | | | | | | |
| *Low category* | Reference | – | Reference | – | Reference | – | Reference | – |
| *Moderate category* | 0.013 (-0.010 – 0.035) | 0.264 | 0.011 (-0.011 – 0.033) | 0.336 | 0.013 (0.001 – 0.025) | **0.040** | 0.012 (-0.001 – 0.024) | 0.061 |
| *High category* | 0.007 (-0.019 – 0.032) | 0.610 | 0.004 (-0.022 – 0.029) | 0.785 | 0.005 (-0.010 – 0.019) | 0.522 | 0.003 (-0.011 – 0.017) | 0.661 |
| **Trunk fat mass index (kg/m$^2$)** | | | | | | | | |
| *Low category* | Reference | – | Reference | – | Reference | – | Reference | – |
| *Moderate category* | 0.003 (-0.019 – 0.026) | 0.763 | 0.002 (-0.021 – 0.024) | 0.875 | 0.008 (-0.005 – 0.020) | 0.234 | 0.007 (-0.006 – 0.019) | 0.284 |
| *High category* | -0.0004 (-0.024 – 0.023) | 0.997 | -0.002 (-0.026 – 0.021) | 0.841 | 0.005 (-0.008 – 0.018) | 0.474 | 0.004 (-0.010 – 0.017) | 0.582 |
| **Lean mass index (kg/m$^2$)** | | | | | | | | |
| *Low category* | Reference | – | Reference | – | Reference | – | Reference | – |
| *Moderate category* | -0.019 (-0.039 – 0.001) | 0.068 | 0.012 (-0.004 – 0.029) | 0.138 | 0.006 (-0.005 – 0.018) | 0.277 | 0.006 (-0.005 – 0.018) | 0.293 |
| *High category* | 0.002 (-0.024 – 0.028) | 0.878 | -0.004 (-0.031 – 0.023) | 0.764 | 0.012 (-0.003 – 0.027) | 0.108 | 0.010 (-0.005 – 0.025) | 0.178 |
| **Body mass index (kg/m$^2$)** | | | | | | | | |
| *Low category* | Reference | – | Reference | – | Reference | – | Reference | – |
| *Moderate category* | 0.005 (-0.015 – 0.024) | 0.654 | 0.004 (-0.009 – 0.017) | 0.538 | -0.005 (-0.016 – 0.006) | 0.417 | -0.002 (-0.010 – 0.006) | 0.639 |
| *High category* | 0.004 (-0.017 – 0.026) | 0.707 | 0.013 (-0.001 – 0.028) | 0.065 | 0.009 (-0.003 – 0.021) | 0.135 | 0.009 (-0.0002 – 0.017) | 0.056 |
| **Systolic blood pressure (mm Hg)** | | | | | | | | |
| *Low category* | Reference | – | Reference | – | Reference | – | Reference | – |
| *Moderate category* | 0.003 (-0.017 – 0.024) | 0.753 | 0.005 (-0.016 – 0.025) | 0.643 | 0.007 (-0.004 – 0.018) | 0.216 | 0.012 (0.004 – 0.020) | **0.003** |
| *High category* | 0.009 (-0.012 – 0.030) | 0.423 | 0.011 (-0.010 – 0.031) | 0.319 | 0.014 (0.003 – 0.025) | **0.014** | 0.021 (0.013 – 0.030) | **<0.0001** |
| **Diastolic blood pressure (mm Hg)** | | | | | | | | |
| *Low category* | Reference | – | Reference | – | Reference | **–** | Reference | **–** |
| *Moderate category* | -0.023 (-0.055 – 0.008) | 0.144 | -0.004 (-0.017 – 0.009) | 0.546 | 0.003 (-0.016 – 0.021) | 0.758 | 0.003 (-0.006 – 0.011) | 0.540 |
| *High category* | -0.003 (-0.036 – 0.030) | 0.874 | 0.017 (0.003 – 0.030) | **0.015** | 0.005 (-0.015 – 0.024) | 0.617 | 0.009 (0.001 – 0.018) | **0.025** |

Effect estimates and CI, confidence interval, from linear mixed model analyses. Associations with p-value <0.05 are considered statistically significant.
Model 1 was adjusted for age at baseline, sex, time, systolic blood pressure, low density lipoprotein, C-reactive protein, fasting blood glucose, and fat mass and/or lean mass depending on the predictor
Model 2 Further adjustment of model 1 for moderate to vigorous physical activity at 24 years, smoking status and family history of hypertension/diabetes/high cholesterol/vascular disease.

**Supplementary Table 9** Cumulative effect of exposure to fat mass, lean mass, and systolic blood pressure from age 9 to 24 years on a 7-year change in carotid-femoral pulse wave velocity and carotid intima-media thickness from age 17 to 24 years (Non-imputed dataset, listwise deletion)

| **N = 301** | **Carotid-femoral pulse wave velocity** | | | | **Carotid intima-media thickness** | | | |
|---|---|---|---|---|---|---|---|---|
| | **Model 1** | | **Model 2** | | **Model 1** | | **Model 2** | |
| | Effect estimate (95% CI) | p-value | Effect estimate (95% CI) | p-value | Effect estimate (95% CI) | p-value | Effect estimate (95% CI) | p-value |
| **Total fat mass (kg)** | | | | | | | | |
| *Low category* | Reference | – | Reference | – | Reference | – | Reference | – |
| *Moderate category* | -0.001 (-0.007 – 0.006) | 0.782 | 0.004 (-0.019 – 0.026) | 0.741 | -0.003 (-0.007 – 0.002) | 0.268 | 0.009 (-0.007 – 0.026) | 0.257 |
| *High category* | 0.001 (-0.006 – 0.008) | 0.765 | 0.001 (-0.023 – 0.025) | 0.932 | -0.002 (-0.008 – 0.003) | 0.365 | 0.009 (-0.009 – 0.026) | 0.331 |
| **Trunk fat mass (kg)** | | | | | | | | |
| *Low category* | Reference | – | Reference | – | Reference | – | Reference | – |
| *Moderate category* | -0.003 (-0.010 – 0.003) | 0.354 | 0.006 (-0.016 – 0.029) | 0.590 | -0.002 (-0.007 – 0.002) | 0.332 | 0.013 (-0.003 – 0.029) | 0.115 |
| *High category* | -0.001 (-0.008 – 0.006) | 0.851 | -0.008 (-0.032 – 0.016) | 0.497 | -0.003 (-0.008 – 0.002) | 0.223 | 0.014 (-0.003 – 0.032) | 0.109 |
| **Lean mass (kg)** | | | | | | | | |
| *Low category* | Reference | – | Reference | – | Reference | – | Reference | – |
| *Moderate category* | -0.004 (-0.011 – 0.002) | 0.169 | 0.010 (-0.013 – 0.030) | 0.438 | 0.009 (0.004 – 0.014) | **<0.0001** | 0.012 (0.0004 – 0.023) | **0.041** |
| *High category* | 0.003 (-0.004 – 0.011) | 0.387 | 0.021 (-0.007 – 0.049) | 0.146 | 0.012 (0.006 – 0.017) | **<0.0001** | 0.015 (-0.0004 – 0.030) | 0.057 |
| **Total fat mass index (kg/m$^2$)** | | | | | | | | |
| *Low category* | Reference | – | Reference | – | Reference | – | Reference | – |
| *Moderate category* | 0.003 (-0.004 – 0.009) | 0.464 | 0.008 (-0.014 – 0.030) | 0.489 | -0.004 (-0.008 – 0.001) | 0.145 | 0.012 (-0.004 – 0.028) | 0.153 |
| *High category* | 0.001 (-0.007 – 0.008) | 0.836 | -0.012 (-0.038 – 0.014) | 0.365 | -0.005 (-0.010 – 0.001) | 0.110 | 0.005 (-0.014 – 0.024) | 0.623 |
| **Trunk fat mass index (kg/m$^2$)** | | | | | | | | |
| *Low category* | Reference | – | Reference | – | Reference | – | Reference | – |
| *Moderate category* | -0.004 (-0.011 – 0.003) | 0.236 | 0.002 (-0.020 – 0.025) | 0.840 | -0.003 (-0.007 – 0.002) | 0.289 | 0.003 (-0.006 – 0.027) | 0.197 |
| *High category* | -0.001 (-0.009 – 0.006) | 0.756 | -0.010 (-0.035 – 0.015) | 0.426 | -0.004 (-0.009 – 0.002) | 0.167 | 0.007 (-0.011 – 0.025) | 0.447 |
| **Lean mass index (kg/m$^2$)** | | | | | | | | |
| *Low category* | Reference | – | Reference | – | Reference | – | Reference | – |
| *Moderate category* | -0.007 (-0.014 – -0.001) | **0.033** | -0.005 (-0.026 – 0.017) | 0.675 | 0.005 (0.001 – 0.010) | **0.016** | -0.006 (-0.023 – 0.011) | 0.497 |
| *High category* | -0.004 (-0.004 – 0.011) | 0.373 | 0.018 (-0.010 – 0.047) | 0.212 | 0.006 (-0.001 – 0.010) | 0.094 | 0.013 (-0.010 – 0.035) | 0.267 |
| **Body mass index (kg/m$^2$)** | | | | | | | | |
| *Low category* | Reference | – | Reference | – | Reference | – | Reference | – |
| *Moderate category* | 0.001 (-0.005 – 0.007) | 0.702 | -0.006 (-0.028 – 0.016) | 0.594 | 0.002 (-0.002 – 0.007) | 0.368 | 0.014 (-0.002 – 0.030) | 0.091 |
| *High category* | -0.004 (-0.011 – 0.003) | 0.281 | -0.010 (-0.033 – 0.014) | 0.415 | 0.002 (-0.003 – 0.007) | 0.423 | 0.004 (-0.014 – 0.021) | 0.667 |
| **Systolic blood pressure (mm Hg)** | | | | | | | | |
| *Low category* | Reference | – | Reference | – | Reference | – | Reference | – |
| *Moderate category* | -0.009 (-0.030 – 0.013) | 0.419 | 0.007 (-0.009 – 0.023) | 0.394 | 0.0002 (-0.015 – 0.016) | 0.974 | 0.014 (0.003 – 0.025) | **0.012** |
| *High category* | 0.008 (-0.014 – 0.030) | 0.410 | 0.020 (0.003 – 0.038) | **0.019** | 0.014 (-0.003 – 0.030) | 0.097 | 0.026 (0.015 – 0.037) | **<0.0001** |
| **Diastolic blood pressure (mm Hg)** | | | | | | | | |
| *Low category* | Reference | – | Reference | – | Reference | – | Reference | – |
| *Moderate category* | 0.027 (0.007 – 0.047) | **0.006** | 0.029 (0.010 – 0.049) | **0.004** | -0.006 (-0.020 – 0.009) | 0.451 | -0.007 (-0.021 – 0.008) | 0.374 |
| *High category* | 0.030 (0.009 – 0.052) | **0.007** | 0.030 (0.009 – 0.052) | **0.007** | -0.011 (-0.027 – 0.005) | 0.187 | -0.011 (-0.028 – 0.005) | 0.167 |

Effect estimates and CI, confidence interval, from linear mixed model analyses. Associations with p-value <0.05 are considered statistically significant.

Model 1 was adjusted for age at baseline, sex, time, systolic blood pressure, low-density lipoprotein, C-reactive protein, fasting blood glucose, and fat mass and/or lean mass depending on the predictor

Model 2 Further adjustment of model 1 for moderate to vigorous physical activity at 24 years, smoking status and family history of hypertension/diabetes/high cholesterol/vascular disease.

**Supplementary Table 10** Descriptive characteristics of participants based on body mass index classification of normal weight and overweight and obesity at age 17 years

| Variables | Male | | | | | Female | | | | |
|---|---|---|---|---|---|---|---|---|---|---|
| | Normal weight | | Overweight and obesity | | P-value | Normal weight | | Overweight and obesity | | P-value |
| | N | Mean (SD) | N | Mean (SD) | | N | Mean (SD) | N | Mean (SD) | |
| ***Anthropometry*** | | | | | | | | | | |
| Height (m) | 1390 | 1.79 (0.07) | 303 | 1.78 (0.07) | 0.438 | 1648 | 1.66 (0.06) | 464 | 1.65 (0.06) | 0.193 |
| *Weight (kg) | 1390 | 66.85 (11.10) | 303 | 87.30 (13.60) | **<0.0001** | 1648 | 57.50 (9.90) | 464 | 75.65 (12.28) | **<0.0001** |
| ***Body composition*** | | | | | | | | | | |
| *Total fat mass (kg) | 1367 | 8.81 (6.54) | 297 | 25.48 (11.85) | **<0.0001** | 1618 | 17.30 (7.04) | 454 | 32.22 (9.97) | **<0.0001** |
| *Trunk fat mass (kg) | 1367 | 4.45 (3.44) | 297 | 14.05 (6.74) | **<0.0001** | 1618 | 8.37 (3.87) | 454 | 16.98 (5.98) | **<0.0001** |
| Lean mass (kg) | 1367 | 54.38 (5.89) | 297 | 57.66 (6.09) | **<0.0001** | 1618 | 37.34 (3.80) | 454 | 40.01 (4.44) | **<0.0001** |
| *Body mass index (kg/m$^2$) | 1390 | 20.99 (2.90) | 303 | 27.11 (3.55) | **<0.0001** | 1648 | 21.18 (3.06) | 454 | 27.48 (4.39) | **<0.0001** |
| ***Metabolic profile*** | | | | | | | | | | |
| Total Cholesterol (mmol/L) | 1030 | 3.51 (0.59) | 227 | 3.77 (0.69) | **<0.0001** | 1013 | 3.93 (0.69) | 278 | 3.97 (0.67) | 0.337 |
| High-density lipoprotein (mmol/L) | 1030 | 1.21 (0.26) | 227 | 1.07 (0.24) | **<0.0001** | 1013 | 1.38 (0.31) | 278 | 1.23 (0.30) | **<0.0001** |
| Low-density lipoprotein (mmol/L) | 1030 | 1.95 (0.53) | 227 | 2.23 (0.64) | **<0.0001** | 1013 | 2.17 (0.62) | 278 | 2.32 (0.63) | **0.001** |
| *Triglyceride (mmol/L) | 1030 | 0.72 (0.32) | 277 | 0.91 (0.53) | **<0.0001** | 1013 | 0.75 (0.35) | 278 | 0.81 (0.44) | **0.001** |
| Glucose (mmol/L) | 1030 | 5.15 (0.75) | 227 | 5.23 (0.38) | 0.145 | 1013 | 4.89 (0.53) | 278 | 4.98 (0.42) | **0.006** |
| *Insulin (mU/L) | 1013 | 5.66 (3.49) | 226 | 8.81 (6.17) | **<0.0001** | 991 | 6.91 (3.99) | 275 | 9.40 (5.85) | **<0.0001** |
| *High sensitivity C-reactive protein (mg/L) | 1030 | 0.39 (0.55) | 227 | 0.77 (1.22) | **<0.0001** | 1013 | 0.60 (1.08) | 278 | 1.10 (2.10) | **<0.0001** |
| ***Vascular measures*** | | | | | | | | | | |
| Pulse rate (beat/mins) | 1388 | 63 (9) | 303 | 64 (10) | **0.021** | 1645 | 67 (10) | 462 | 69 (10) | **0.008** |
| Systolic blood pressure (mm Hg) | 1388 | 119 (9) | 303 | 124 (9) | **<0.0001** | 1645 | 109 (7) | 462 | 114 (8) | **<0.0001** |
| Diastolic blood pressure (mm Hg) | 1388 | 63 (6) | 303 | 66 (7) | **<0.0001** | 1645 | 64 (6) | 462 | 68 (7) | **<0.0001** |
| *Carotid-femoral PWV (m/s) | 1390 | 5.95 (0.85) | 303 | 6.01 (0.78) | 0.853 | 1648 | 5.45 (0.74) | 464 | 5.56 (0.73) | **0.006** |
| *Carotid IMT (mm) | 1390 | 0.48 (0.06) | 303 | 0.48 (0.06) | 0.996 | 1648 | 0.47 (0.05) | 464 | 0.47 (0.07) | 0.346 |

The values are means (standard deviations) and *median (interquartile range). Differences were tested using Student's t-test for normally distributed continuous variables and Mann–Whitney U test for skewed continuous variables. A 2-sided P-value <0.05 is considered statistically significant. Body mass index was classified as <24.99 as normal weight and >24.99 as overweight and obese.

**Supplementary Table 11** Descriptive characteristics of participants based on body mass index classification of normal weight and overweight and obesity at age 24 years

| Variables | Male | | | | | Female | | | | |
|---|---|---|---|---|---|---|---|---|---|---|
| | Normal weight | | Overweight and obesity | | P-value | Normal weight | | Overweight and obesity | | P-value |
| | N | Mean (SD) | N | Mean (SD) | | N | Mean (SD) | N | Mean (SD) | |
| ***Anthropometry*** | | | | | | | | | | |
| Height (m) | 571 | 1.81 (0.07) | 377 | 1.79 (0.07) | **0.006** | 933 | 1.66 (0.06) | 550 | 1.66 (0.06) | 0.131 |
| *Weight (kg) | 571 | 71.70 (11.30) | 377 | 89.0 (13.80) | **<0.0001** | 933 | 60.40 (8.75) | 550 | 79.30 (16.45) | **<0.0001** |
| ***Body composition*** | | | | | | | | | | |
| *Total fat mass (kg) | 554 | 14.72 (5.66) | 371 | 25.99 (11.51) | **<0.0001** | 907 | 18.49 (5.98) | 526 | 32.71 (12.54) | **<0.0001** |
| *Trunk fat mass (kg) | 554 | 6.94 (3.44) | 371 | 13.57 (7.26) | **<0.0001** | 907 | 7.94 (3.30) | 526 | 16.06 (7.22) | **<0.0001** |
| Lean mass (kg) | 554 | 53.93 (6.20) | 371 | 60.77 (6.83) | **<0.0001** | 907 | 39.36 (4.10) | 526 | 44.61 (5.05) | **<0.0001** |
| *Body mass index (kg/m$^2$) | 571 | 22.17 (2.65) | 377 | 27.32 (3.63) | **<0.0001** | 933 | 21.88 (2.92) | 550 | 28.54 (5.47) | **<0.0001** |
| ***Metabolic profile*** | | | | | | | | | | |
| Total Cholesterol (mmol/L) | 510 | 4.19 (0.81) | 324 | 4.60 (0.84) | **<0.0001** | 753 | 4.44 (0.84) | 407 | 4.55 (0.78) | **0.023** |
| High-density lipoprotein (mmol/L) | 510 | 1.49 (0.37) | 324 | 1.28 (0.33) | **<0.0001** | 753 | 1.75 (0.41) | 407 | 1.49 (0.40) | **<0.0001** |
| Low-density lipoprotein (mmol/L) | 509 | 2.29 (0.74) | 323 | 2.74 (0.80) | **<0.0001** | 753 | 2.30 (0.72) | 407 | 2.57 (0.74) | **<0.0001** |
| *Triglyceride (mmol/L) | 509 | 0.82 (0.42) | 324 | 1.10 (0.75) | **<0.0001** | 753 | 0.76 (0.39) | 407 | 0.93 (0.52) | **<0.0001** |
| Glucose (mmol/L) | 510 | 5.44 (0.65) | 324 | 5.50 (0.44) | 0.138 | 753 | 5.14 (0.48) | 407 | 5.36 (0.83) | **<0.0001** |
| *Insulin (mU/L) | 510 | 6.16 (3.37) | 324 | 9.71 (8.42) | **<0.0001** | 753 | 6.65 (3.74) | 407 | 11.28 (7.70) | **<0.0001** |
| *High sensitivity C-reactive protein (mg/L) | 430 | 0.49 (0.77) | 315 | 0.95 (1.44) | **<0.0001** | 691 | 0.75 (1.57) | 396 | 1.81 (2.96) | **<0.0001** |
| ***Vascular measures*** | | | | | | | | | | |
| Pulse rate (beat/mins) | 570 | 63 (9) | 376 | 66 (11) | **<0.0001** | 930 | 68 (10) | 548 | 70 (10) | **<0.0001** |
| Systolic blood pressure (mm Hg) | 570 | 120 (10) | 376 | 126 (10.2) | **<0.0001** | 930 | 109 (9) | 548 | 116 (9) | **<0.0001** |
| Diastolic blood pressure (mm Hg) | 570 | 66 (7) | 376 | 70 (8) | **<0.0001** | 930 | 64 (7) | 548 | 70 (8) | **<0.0001** |
| *Carotid-femoral PWV (m/s) | 387 | 6.53 (1.30) | 250 | 6.40 (1.03) | 0.134 | 635 | 5.87 (1.03) | 368 | 5.97 (1.0) | **0.004** |
| *Carotid IMT (mm) | 330 | 0.46 (0.07) | 201 | 0.47 (0.07) | 0.159 | 555 | 0.45 (0.06) | 305 | 0.45 (0.06) | 0.549 |

The values are means (standard deviations) and *median (interquartile range). Differences were tested using Student's t-test for normally distributed continuous variables and Mann–Whitney U test for skewed continuous variables. A 2-sided P-value <0.05 is considered statistically significant. Body mass index was classified as <24.99 as normal weight and >24.99 as overweight and obese.

**Supplementary Table 12** Descriptive characteristics based on systolic blood pressure classification as normotensive or elevated blood pressure and hypertension at age 17 years

| | Male | | | | | Female | | | | |
|---|---|---|---|---|---|---|---|---|---|---|
| **Variables** | **Normotensive** | | **Elevated blood pressure** | | **P-value** | **Normotensive** | | **Elevated blood pressure** | | **P-value** |
| | N | Mean (SD) | N | Mean (SD) | | N | Mean (SD) | N | Mean (SD) | |
| ***Anthropometry*** | | | | | | | | | | |
| Height (m) | 896 | 1.78 (0.07) | 795 | 1.80 (0.07) | **<0.0001** | 1918 | 1.65 (0.06) | 189 | 1.66 (0.06) | 0.064 |
| *Weight (kg) | 899 | 66.30 (13.80) | 795 | 72.20 (14.30) | **<0.0001** | 1920 | 59.70 (12.70) | 189 | 69.70 (19.60) | **<0.0001** |
| ***Body composition*** | | | | | | | | | | |
| *Total fat mass (kg) | 885 | 8.94 (8.31) | 788 | 12.05 (11.15) | **<0.0001** | 1887 | 18.97 (9.74) | 188 | 26.55 (16.66) | **<0.0001** |
| *Trunk fat mass (kg) | 885 | 4.49 (4.36) | 788 | 6.27 (6.31) | **<0.0001** | 1887 | 9.23 (5.24) | 188 | 13.30 (9.26) | **<0.0001** |
| Lean mass (kg) | 885 | 53.57 (5.75) | 788 | 56.56 (5.99) | **<0.0001** | 1887 | 37.73 (3.96) | 188 | 39.85 (4.96) | **<0.0001** |
| *Body mass index (kg/m$^2$) | 896 | 20.83 (3.28) | 795 | 22.48 (4.24) | **<0.0001** | 1918 | 21.81 (4.16) | 189 | 25.28 (7.62) | **<0.0001** |
| ***Metabolic profile*** | | | | | | | | | | |
| Total Cholesterol (mmol/L) | 673 | 3.52 (0.59) | 602 | 3.60 (0.64) | **0.036** | 1194 | 3.93 (0.69) | 114 | 3.96 (0.64) | 0.654 |
| High-density lipoprotein (mmol/L) | 673 | 1.19 (0.25) | 602 | 1.18 (0.27) | 0.907 | 1194 | 1.36 (0.32) | 114 | 1.27 (0.30) | **0.007** |
| Low-density lipoprotein (mmol/L) | 673 | 1.98 (0.55) | 602 | 2.01 (0.57) | 0.325 | 1194 | 2.20 (0.64) | 114 | 2.28 (0.54) | 0.171 |
| *Triglyceride (mmol/L) | 673 | 0.71 (0.32) | 602 | 0.78 (0.39) | **<0.0001** | 1194 | 0.75 (0.37) | 114 | 0.85 (0.42) | **0.008** |
| Glucose (mmol/L) | 673 | 5.15 (0.53) | 602 | 5.19 (0.84) | 0.282 | 1194 | 4.90 (0.51) | 114 | 4.98 (0.50) | 0.143 |
| *Insulin (mU/L) | 666 | 5.72 (3.76) | 591 | 6.43 (4.37) | **<0.0001** | 1171 | 7.26 (4.25) | 112 | 8.32 (7.73) | **<0.0001** |
| *High sensitivity C-reactive protein (mg/L) | 673 | 0.39 (0.61) | 602 | 0.51 (0.81) | **0.001** | 1194 | 0.65 (1.23) | 114 | 0.86 (1.74) | **0.003** |
| ***Vascular measures*** | | | | | | | | | | |
| Pulse rate (beat/mins) | 908 | 62 (9) | 809 | 64 (10) | **<0.0001** | 1941 | 67 (10) | 197 | 71 (11) | **<0.0001** |
| Systolic blood pressure (mm Hg) | 908 | 113 (5) | 809 | 127 (6) | **<0.0001** | 1941 | 109 (6) | 197 | 125 (5) | **<0.0001** |
| Diastolic blood pressure (mm Hg) | 908 | 61 (5) | 809 | 66 (6) | **<0.0001** | 1941 | 64 (5) | 197 | 73 (7) | **<0.0001** |
| *Carotid-femoral PWV (m/s) | 908 | 5.87 (0.86) | 809 | 6.07 (0.78) | **<0.0001** | 1941 | 5.44 (0.73) | 197 | 5.83 (0.82) | **<0.0001** |
| *Carotid IMT (mm) | 908 | 0.48 (0.06) | 809 | 0.48 (0.06) | **0.018** | 1941 | 0.47 (0.06) | 197 | 0.47 (0.07) | 0.116 |

The values are means (standard deviations) and *median (interquartile range). Differences were tested using Student's t-test for normally distributed continuous variables and Mann–Whitney U test for skewed continuous variables. A 2-sided P-value <0.05 is considered statistically significant. Systolic blood pressure was classified as <120 mmHg as normotensive and >120 mmHg as elevated blood pressure and hypertension.

**Supplementary Table S13** Descriptive characteristics based on systolic blood pressure classification as normotensive or elevated blood pressure and hypertension at age 24 years

| | Male | | | | | Female | | | | |
|---|---|---|---|---|---|---|---|---|---|---|
| **Variables** | **Normotensive** | | **Elevated blood pressure** | | **P-value** | **Normotensive** | | **Elevated blood pressure** | | **P-value** |
| | N | Mean (SD) | N | Mean (SD) | | N | Mean (SD) | N | Mean (SD) | |
| ***Anthropometry*** | | | | | | | | | | |
| Height (m) | 421 | 1.79 (0.07) | 526 | 1.81 (0.07) | **0.007** | 1212 | 1.66 (0.06) | 267 | 1.67 (0.06) | **0.023** |
| *Weight (kg) | 421 | 73.10 (16.60) | 525 | 80.80 (15.85) | **<0.0001** | 1213 | 63.50 (14.65) | 266 | 74.25 (22.60) | **<0.0001** |
| ***Body composition*** | | | | | | | | | | |
| *Total fat mass (kg) | 411 | 15.65 (9.35) | 515 | 19.70 (10.91) | **<0.0001** | 1178 | 21.15 (10.43) | 255 | 28.46 (16.85) | **<0.0001** |
| *Trunk fat mass (kg) | 411 | 7.40 (5.33) | 515 | 9.97 (6.71) | **<0.0001** | 1178 | 9.41 (5.85) | 255 | 13.12 (9.42) | **<0.0001** |
| Lean mass (kg) | 411 | 54.90 (7.10) | 515 | 58.12 (7.12) | **<0.0001** | 1178 | 40.78 (4.88) | 255 | 43.72 (5.56) | **<0.0001** |
| *Body mass index (kg/m$^2$) | 421 | 22.75 (4.43) | 525 | 24.74 (4.78) | **<0.0001** | 1212 | 23.02 (4.82) | 266 | 26.95 (7.90) | **<0.0001** |
| ***Metabolic profile*** | | | | | | | | | | |
| Total Cholesterol (mmol/L) | 375 | 4.30 (0.84) | 464 | 4.38 (0.85) | 0.149 | 975 | 4.46 (0.83) | 194 | 4.57 (0.75) | 0.073 |
| High-density lipoprotein (mmol/L) | 375 | 1.45 (0.36) | 464 | 1.37 (0.37) | **0.001** | 975 | 1.68 (0.42) | 194 | 1.54 (0.44) | **<0.0001** |
| Low-density lipoprotein (mmol/L) | 374 | 2.41 (0.79) | 463 | 2.50 (0.79) | 0.073 | 975 | 2.37 (0.74) | 194 | 2.56 (0.73) | **0.001** |
| *Triglyceride (mmol/L) | 374 | 0.82 (0.48) | 464 | 0.94 (0.58) | **0.001** | 975 | 0.79 (0.43) | 194 | 0.93 (0.54) | **<0.0001** |
| Glucose (mmol/L) | 375 | 5.47 (0.71) | 464 | 5.46 (0.46) | 0.684 | 975 | 5.21 (0.59) | 194 | 5.28 (0.81) | 0.188 |
| *Insulin (mU/L) | 375 | 6.35 (4.46) | 464 | 7.52 (5.59) | **<0.0001** | 975 | 7.35 (5.02) | 194 | 10.07 (7.80) | **<0.0001** |
| *High sensitivity C-reactive protein (mg/L) | 322 | 0.61 (0.95) | 428 | 0.68 (1.24) | **0.014** | 907 | 0.92 (1.90) | 188 | 1.40 (3.25) | **<0.0001** |
| ***Vascular measures*** | | | | | | | | | | |
| Pulse rate (beat/mins) | 424 | 63 (10) | 528 | 65 (11) | 0.122 | 1226 | 68 (10) | 269 | 72 (10) | **<0.0001** |
| Systolic blood pressure (mm Hg) | 424 | 114 (5) | 528 | 130 (8) | **<0.0001** | 1226 | 108 (7) | 269 | 126 (5) | **<0.0001** |
| Diastolic blood pressure (mm Hg) | 424 | 63 (6) | 528 | 70 (8) | **<0.0001** | 1226 | 65 (6) | 269 | 75 (8) | **<0.0001** |
| *Carotid-femoral PWV (m/s) | 296 | 6.36 (1.17) | 344 | 6.53 (1.18) | **0.019** | 837 | 5.90 (1.0) | 175 | 6.15 (1.12) | **<0.0001** |
| *Carotid IMT (mm) | 253 | 0.45 (0.06) | 279 | 0.47 (0.06) | **<0.0001** | 720 | 0.45 (0.06) | 146 | 0.46 (0.06) | **0.012** |

The values are means (standard deviations) and *median (interquartile range). Differences were tested using Student's t-test for normally distributed continuous variables and Mann–Whitney U test for skewed continuous variables. A 2-sided P-value <0.05 is considered statistically significant. Systolic blood pressure was classified as <120 mmHg as normotensive and >120 mmHg as elevated blood pressure and hypertension.

**Supplementary Table 14** Cumulative effect of exposure to fat mass, lean mass, and systolic blood pressure from age 9 to 24 years on a 7-year change in carotid-femoral pulse wave velocity and carotid intima-media thickness from age 17 to 24 years based on body mass index status at 24 years of normal weight and overweight and obese

| | **Carotid-femoral pulse wave velocity** | | | | **Carotid intima-media thickness** | | | |
|---|---|---|---|---|---|---|---|---|
| | **Normal weight** | | **Overweight and obesity** | | **Normal weight** | | **Overweight and obesity** | |
| | Effect estimate (95% CI) | p-value | Effect estimate (95% CI) | p-value | Effect estimate (95% CI) | p-value | Effect estimate (95% CI) | p-value |
| **Total fat mass (kg)** | | | | | | | | |
| *Low category* | Reference | – | Reference | – | Reference | – | Reference | – |
| *High category* | 0.007 (-0.009 – 0.024) | 0.365 | -0.015 (-0.043 – 0.013) | 0.280 | -0.011 (-0.022 – -0.0003) | **0.044** | 0.001 (-0.017 – 0.019) | 0.945 |
| **Trunk fat mass (kg)** | | | | | | | | |
| *Low category* | Reference | – | Reference | – | Reference | – | Reference | – |
| *High category* | 0.008 (-0.006 – 0.021) | 0.252 | -0.019 (-0.046 – 0.008) | 0.171 | -0.013 (-0.024 – -0.002) | **0.027** | -0.001 (-0.019 – 0.016) | 0.872 |
| **Lean mass (kg)** | | | | | | | | |
| *Low category* | Reference | – | Reference | – | Reference | – | Reference | – |
| *High category* | 0.015 (0.001 – 0.028) | **0.036** | -0.001 (-0.018 – 0.016) | 0.901 | 0.012 (0.001 – 0.023) | **0.030** | 0.011 (-0.003 – 0.026) | 0.128 |
| **Body mass index (kg/m$^2$)** | | | | | | | | |
| *Low category* | Reference | – | Reference | – | Reference | – | Reference | – |
| *High category* | -0.001 (-0.015 – 0.014) | 0.933 | -0.005 (-0.035 – 0.024) | 0.734 | -0.001 (-0.013 – 0.012) | 0.943 | -0.009 (-0.037 – 0.019) | 0.522 |
| **Systolic blood pressure (mm Hg)** | | | | | | | | |
| *Low category* | Reference | – | Reference | – | Reference | – | Reference | – |
| *High category* | 0.008 (-0.004 – 0.019) | 0.170 | 0.022 (0.005 – 0.039) | **0.012** | 0.012 (0.005 – 0.020) | **0.001** | 0.008 (-0.004 – 0.021) | 0.183 |

Effect estimates and CI, confidence interval, from linear mixed model analyses. Associations with p-value <0.05 are considered statistically significant. Normal weight was classified as body mass index <24.99 kg/m$^2$ while overweight and obesity was based on >24.99 kg/m$^2$.

Model was adjusted for age at baseline, sex, time, systolic blood pressure, low-density lipoprotein, C-reactive protein, fasting blood glucose, and fat mass and/or lean mass depending on the predictor, moderate to vigorous physical activity at 24 years, smoking status and family history of hypertension/diabetes/high cholesterol/vascular disease.

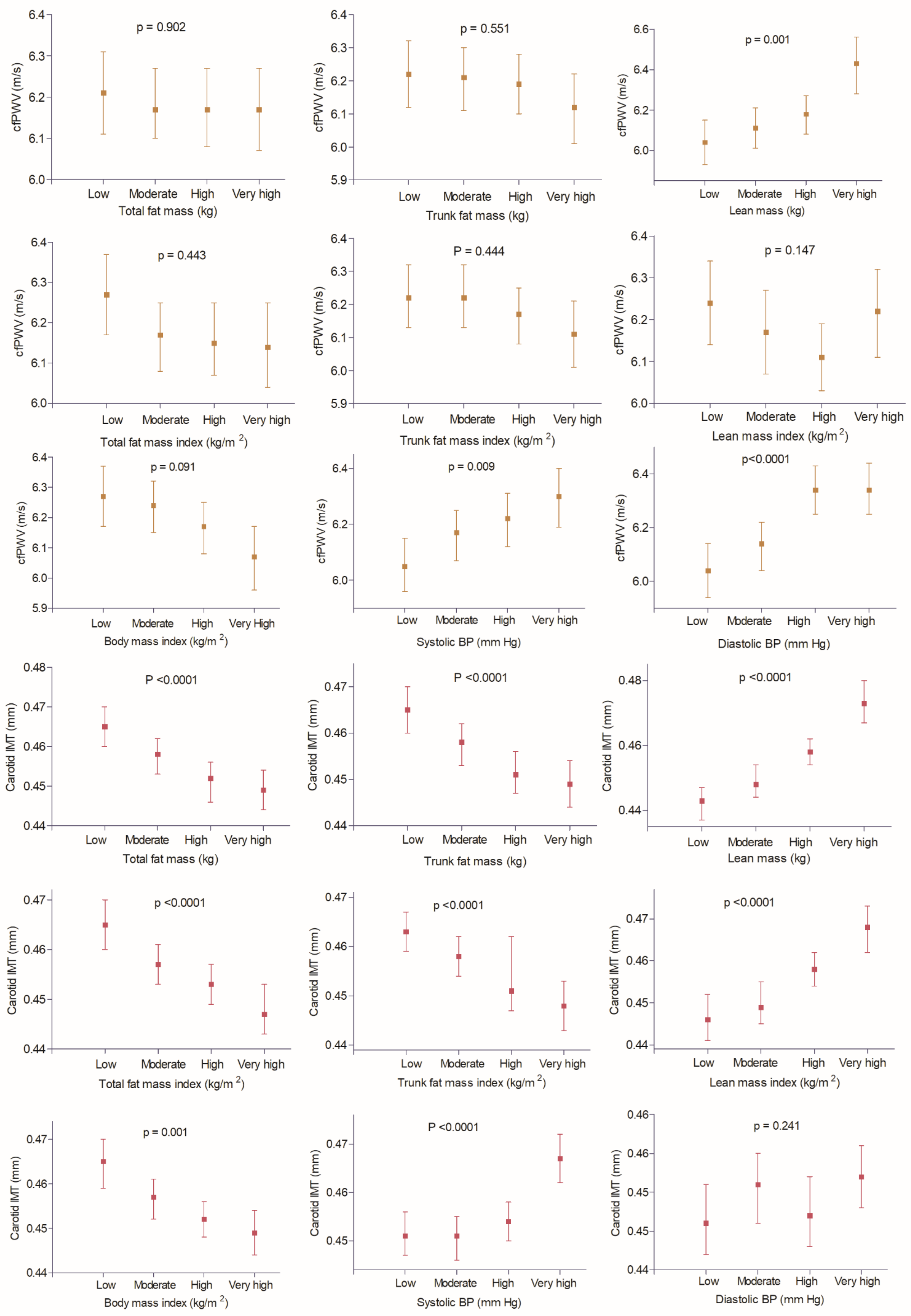
p = 0.902
cfPWV (m/s)
Low Moderate High Very high
Total fat mass (kg)
p = 0.551
Trunk fat mass (kg)
p = 0.001
Lean mass (kg)
p = 0.443
Total fat mass index (kg/m 2)
P = 0.444
Trunk fat mass index (kg/m 2)
p = 0.147
Lean mass index (kg/m 2)
p = 0.091
Body mass index (kg/m 2)
p = 0.009
Systolic BP (mm Hg)
p<0.0001
Diastolic BP (mm Hg)
Carotid IMT (mm)
P <0.0001
Total fat mass (kg)
P <0.0001
Trunk fat mass (kg)
p <0.0001
Lean mass (kg)
p <0.0001
Total fat mass index (kg/m 2)
p <0.0001
Trunk fat mass index (kg/m 2)
p <0.0001
Lean mass index (kg/m 2)
p = 0.001
Body mass index (kg/m 2)
P <0.0001
Systolic BP (mm Hg)
p = 0.241
Diastolic BP (mm Hg)

**Supplementary Figure 1** Inter-quartile differences in the cross-sectional associations of categories of fat mass, lean mass and blood pressure with carotid-femoral pulse wave velocity and carotid intima-media thickness at age 24 years.
cfPWV, carotid-femoral pulse wave velocity; Carotid IMT, carotid intima-media thickness.

General linear model multivariable analyses were adjusted for age, sex, low-density lipoprotein, C-reactive protein, fasting blood glucose, systolic blood pressure and fat mass and/or lean mass depending on the predictor, moderate to vigorous physical activity, smoking status and family history of hypertension/diabetes/high cholesterol/vascular disease. Predictors were skewed variables which were logarithmically transformed before categorizing into age- and population-specific quartiles. Outcome variables were skewed and logarithmically transformed. Mean differences and confidence intervals were back-transformed and represent the mean percentage difference in outcome variables. The p-value is for differences across the groups. All predictors and outcomes had complete case (n=1799) but multiple imputations were used to account for missing covariates. Quartile 1 is Low, Quartile 2 is Moderate, Quartile 3 is High and Quartile 4 is Very High.

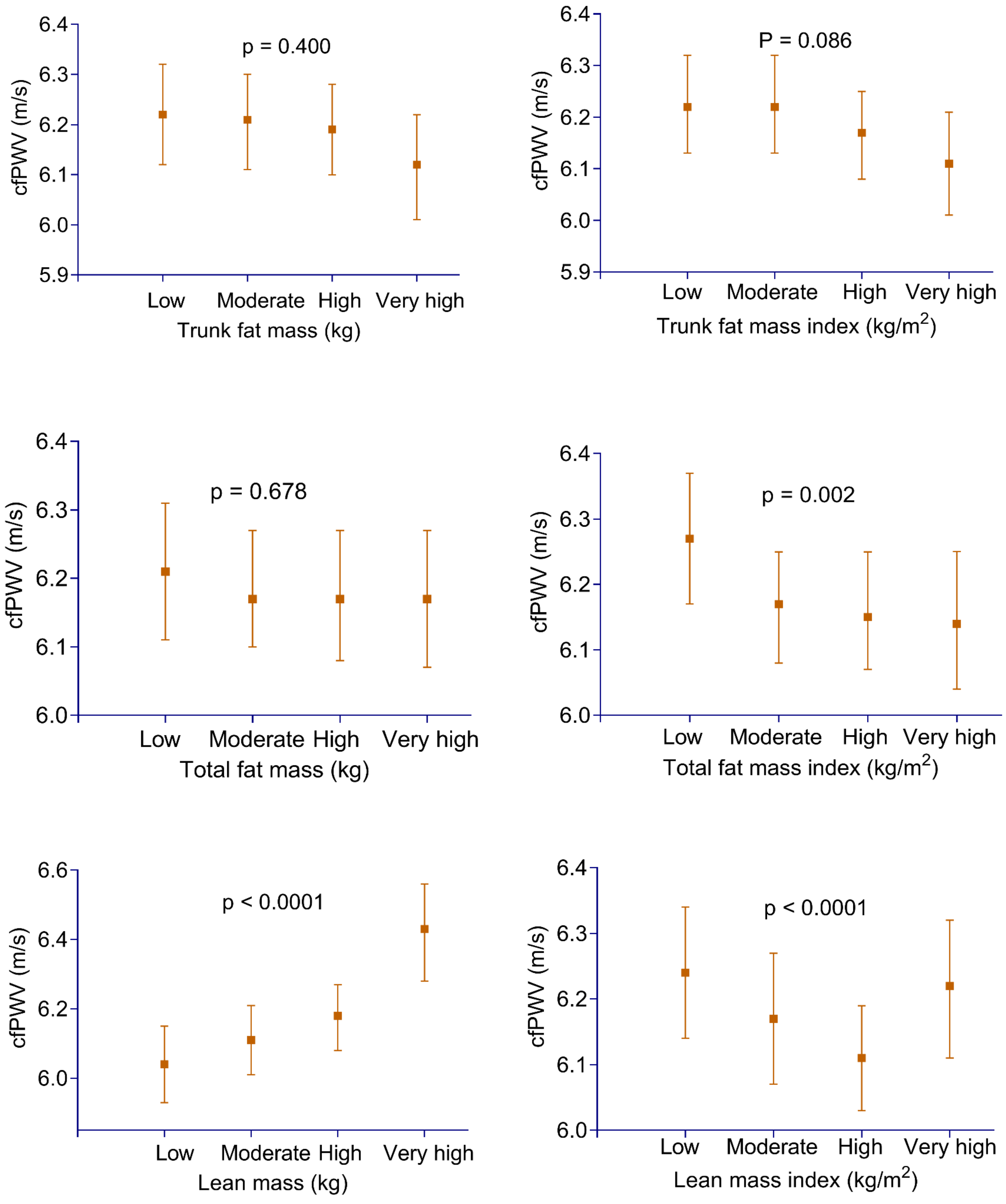
p = 0.400
cfPWV (m/s)
6.4
6.3
6.2
6.1
6.0
5.9
Low
Moderate
High
Very high
Trunk fat mass (kg)
P = 0.086
cfPWV (m/s)
6.4
6.3
6.2
6.1
6.0
5.9
Low
Moderate
High
Very high
Trunk fat mass index (kg/m2)
p = 0.678
cfPWV (m/s)
6.4
6.3
6.2
6.1
6.0
Low
Moderate
High
Very high
Total fat mass (kg)
p = 0.002
cfPWV (m/s)
6.4
6.3
6.2
6.1
6.0
Low
Moderate
High
Very high
Total fat mass index (kg/m2)
p < 0.0001
cfPWV (m/s)
6.6
6.4
6.2
6.0
Low
Moderate
High
Very high
Lean mass (kg)
p < 0.0001
cfPWV (m/s)
6.4
6.3
6.2
6.1
6.0
Low
Moderate
High
Very high
Lean mass index (kg/m2)

**Supplementary Figure 2 Linear trend in the cross-sectional associations of categories of total fat mass, trunk fat mass, and lean mass with carotid-femoral pulse wave velocity at age 24 years.**

All multivariable analyses were adjusted for age, sex, low-density lipoprotein, C-reactive protein, fasting blood glucose, systolic blood pressure, and fat mass and/or lean mass depending on the predictor, moderate to vigorous physical activity, smoking status, and family history of hypertension/diabetes/high cholesterol/vascular disease. Predictors were skewed variables which were logarithmically transformed before categorizing into age- and population-specific quartiles. Outcome variables were skewed and logarithmically transformed. Mean differences and confidence intervals were back-transformed and represent the mean percentage difference in outcome variables. The p-value is for linear trend. All predictors and outcomes had complete cases (n=1799) but multiple imputations were used to account for missing covariates. cfPWV, carotid-femoral pulse wave velocity. Quartile 1 is Low, Quartile 2 is Moderate, Quartile 3 is High, and Quartile 4 is Very High.

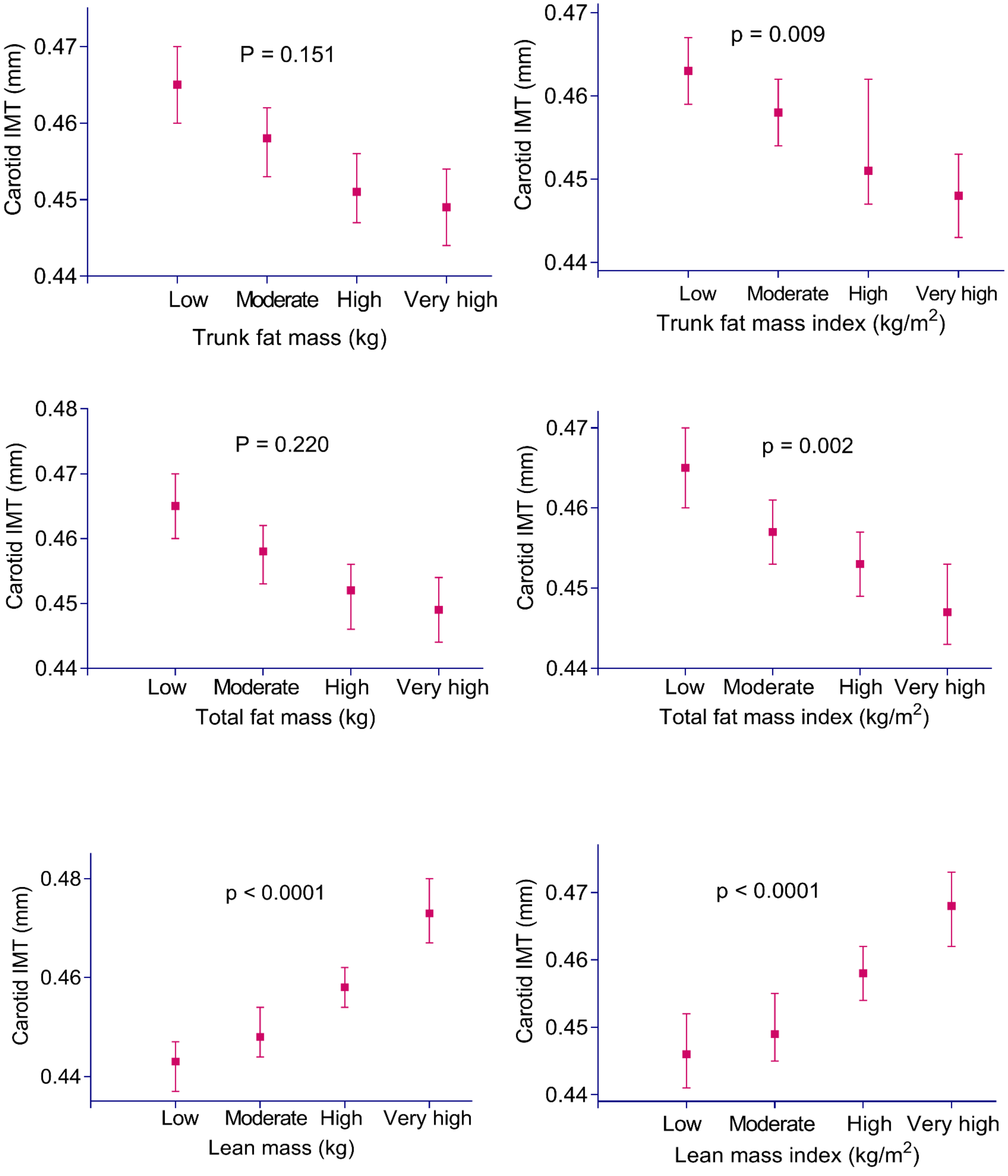
Carotid IMT (mm)
0.47
0.46
0.45
0.44
P = 0.151
Low
Moderate
High
Very high
Trunk fat mass (kg)
p = 0.009
Trunk fat mass index (kg/m2)
0.48
P = 0.220
Total fat mass (kg)
p = 0.002
Total fat mass index (kg/m2)
p < 0.0001
Lean mass (kg)
Lean mass index (kg/m2)

**Supplementary Figure 3 Linear trend in the cross-sectional associations of categories of trunk fat mass, total fat mass and lean mass with carotid intima-media thickness at age 24 years.**

All multivariable analyses were adjusted for age, sex, low-density lipoprotein, C-reactive protein, fasting blood glucose, systolic blood pressure, and fat mass and/or lean mass depending on the predictor, moderate to vigorous physical activity, smoking status, and family history of hypertension/diabetes/high cholesterol/vascular disease. Predictors were skewed variables which were logarithmically transformed before categorizing into age- and population-specific quartiles. Outcome variables were skewed and logarithmically transformed. Mean differences and confidence intervals were back-transformed and represent the mean percentage difference in outcome variables. The p-value is for linear trend. All predictors and outcomes had complete cases (n=1799) but multiple imputations were used to account for missing covariates. Carotid IMT, carotid intima-media thickness. Quartile 1 is Low, Quartile 2 is Moderate, Quartile 3 is High, and Quartile 4 is Very High.

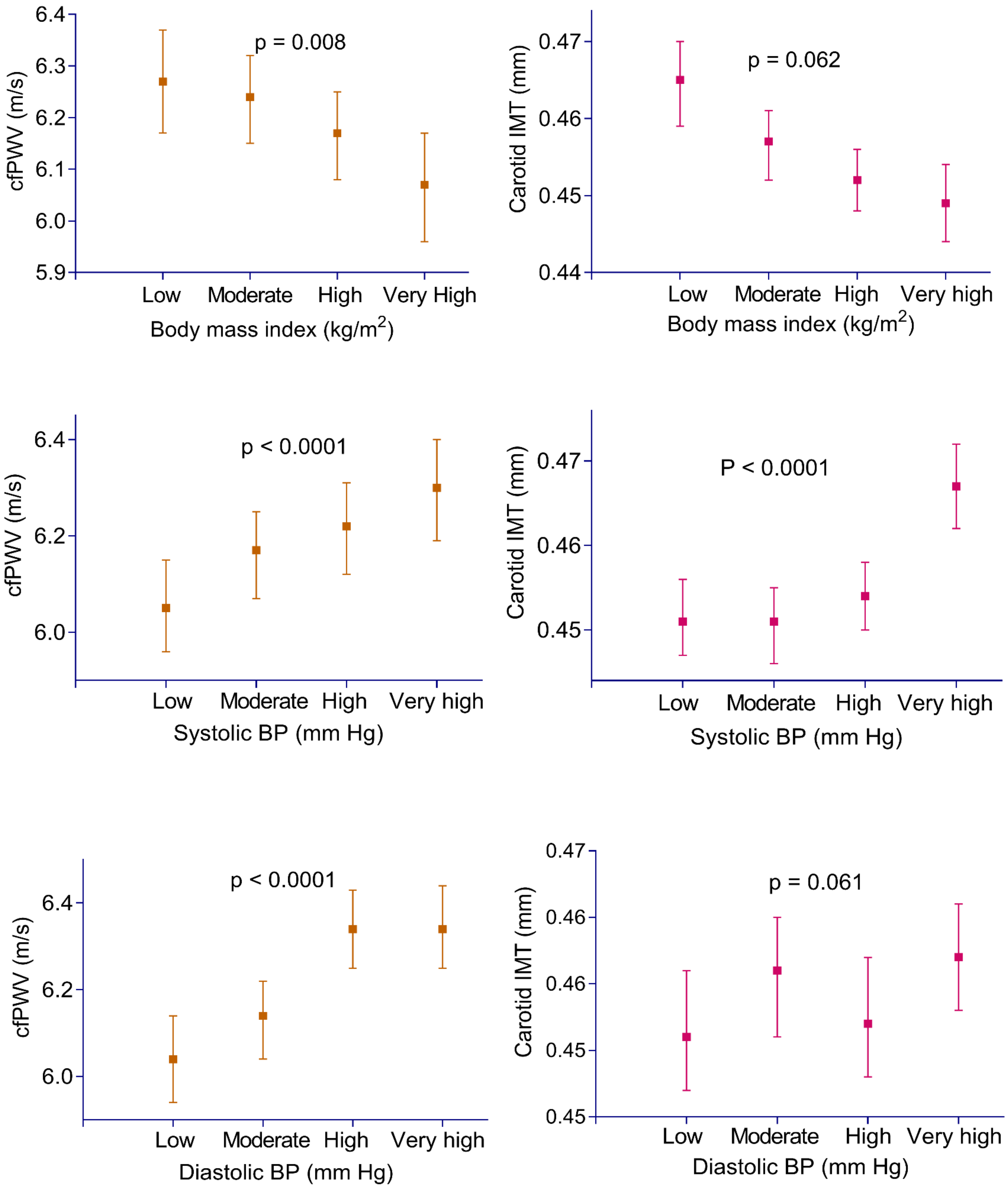
cfPWV (m/s)
6.4
6.3
6.2
6.1
6.0
5.9
p = 0.008
Low
Moderate
High
Very High
Body mass index (kg/m2)
Carotid IMT (mm)
0.47
0.46
0.45
0.44
p = 0.062
Low
Moderate
High
Very high
Body mass index (kg/m2)
cfPWV (m/s)
6.4
6.2
6.0
p < 0.0001
Low
Moderate
High
Very high
Systolic BP (mm Hg)
Carotid IMT (mm)
0.47
0.46
0.45
P < 0.0001
Low
Moderate
High
Very high
Systolic BP (mm Hg)
cfPWV (m/s)
6.4
6.2
6.0
p < 0.0001
Low
Moderate
High
Very high
Diastolic BP (mm Hg)
Carotid IMT (mm)
0.47
0.46
0.46
0.45
0.45
p = 0.061
Low
Moderate
High
Very high
Diastolic BP (mm Hg)

**Supplementary Figure 4 Linear trend in the cross-sectional associations of categories of body mass index, systolic and diastolic blood pressure with carotid-femoral pulse wave velocity and carotid intima-media thickness at age 24 years.**

All multivariable analyses were adjusted for age, sex, low-density lipoprotein, C-reactive protein, fasting blood glucose, systolic blood pressure, and fat mass and/or lean mass depending on the predictor, moderate to vigorous physical activity, smoking status, and family history of hypertension/diabetes/high cholesterol/vascular disease. Predictors were skewed variables which were logarithmically transformed before categorizing into age- and population-specific quartiles. Outcome variables were skewed and logarithmically transformed. Mean differences and confidence intervals were back-transformed and represent the mean percentage difference in outcome variables. The p-value is for linear trend. All predictors and outcomes had complete cases (n=1799) but multiple imputations were used to account for missing covariates. cfPWV, carotid-femoral pulse wave velocity; Carotid IMT, carotid intima-media thickness. Quartile 1 is Low, Quartile 2 is Moderate, Quartile 3 is High, and Quartile 4 is Very High.
